# Dual-Enzyme Biocatalytic Synthesis of Novel Rebaudioside M Analogs with Enhanced Aqueous Solubility, Physicochemical Stability, and Sensory Profiles

**DOI:** 10.3390/foods15152647

**Published:** 2026-07-28

**Authors:** Hye-Jin Kang, Ok-Cheol Kim, Da-Eun Jang, Hee-Min Lee, Bu-Soo Park, Masayuki Okuyama, Jeong-Yong Cho, Seong-Jin Hong, Young-Min Kim

**Affiliations:** 1 Research Institute of Agricultural Science and Technology, Chonnam National University, Gwangju 61186, Republic of Korea; jyhj8818@gmail.com; 2 Department of Integrative Food, Bioscience and Biotechnology, Graduate School of Chonnam National University, Gwangju 61186, Republic of Korea; okcheol9901@naver.com (O.-C.K.); wkdekdms2686@naver.com (D.-E.J.); jyongcho17@chonnam.ac.kr (J.-Y.C.); 3 Industrial Intelligence Research Division, World Institute of Kimchi, Gwangju 61755, Republic of Korea; hmlee@wikim.re.kr; 4 Food Biotech R&D Center, Samyang Corp., Seongnam-si 13488, Republic of Korea; busoo.park@samyang.com; 5 Research Faculty of Agriculture, Hokkaido University, Sapporo 060-8589, Japan; okuyama@agr.hokudai.ac.jp

**Keywords:** steviol glycosides, stevioside, rebaudioside M, glucosyltransferase, *Leuconostoc kimchii*, UGT76G1, solubility enhancement

## Abstract

Rebaudioside M (RM), a steviol glycoside derived from *Stevia rebaudiana* Bertoni, has emerged as a promising next-generation zero-calorie sweetener. However, its low natural abundance and poor aqueous solubility limit its practical application in beverage formulations. To address these limitations, two novel RM analogs, RMLC1 and RMLC2, were synthesized from stevioside (ST) through a two-step biocatalytic strategy. ST was first α-glucosylated by *Leuconostoc kimchii* dextransucrase to generate the α-glucosylated intermediate RD2, which was subsequently β-glucosylated by UGT76G1 to produce RMLC1 and RMLC2, with more than 95% conversion of RD2. RMLC1 and RMLC2 were stable at temperatures up to 120 °C and across a pH range of 2–10. RMLC1 and RMLC2 exhibited 139- and 127-fold higher aqueous solubility than RM, respectively, due to α-glucosylation-induced changes in molecular conformation. In a commercial low-pH beverage, both analogs showed approximately 3-fold greater storage stability than RM. In vitro digestion assays demonstrated that both analogs released less than half the glucose generated from enzymatically modified stevia. Electronic tongue analysis suggested that RMLC1 exhibited a balanced, blend-friendly taste profile, while RMLC2 displayed a sweetness-forward profile with minimal bitterness.

## 1. Introduction

Steviol glycosides (SGs) are a class of diterpenoid compounds that constitute the principal natural sweeteners in *Stevia rebaudiana* [1]. Given their high sweetness intensity and negligible caloric value, the major SGs, such as stevioside (ST) and rebaudioside A (RA), have been widely adopted as sugar substitutes in the food and beverage industries. However, the minor components rebaudioside D (RD) and rebaudioside M (RM) (approximately 0.4–0.5% [*w*/*w*] of the total dry leaf weight) exhibit higher sweetness intensity and significantly lower lingering bitterness than ST and RA [2,3]. Among these, RM is particularly attractive owing to its clean, sucrose-like sweetness profile that most closely resembles that of sucrose among all known SGs [4]. Nevertheless, the extremely low natural abundance of RM renders its direct extraction economically infeasible, necessitating alternative production approaches.

To address the low natural abundance of RM, current production strategies mainly include microbial de novo biosynthesis and enzymatic bioconversion from more abundant SGs, such as ST and RA. A representative microbial route involves de novo biosynthesis in *Saccharomyces cerevisiae*, enabling heterologous reconstruction of the SG biosynthetic pathway for direct RM production. Although this approach is conceptually promising, the reported yield remains relatively low at 67 mg/L, restricting its industrial application [5]. Alternatively, in vitro biocatalytic transformation using glycosyltransferases has emerged as a practical strategy because of its high conversion efficiency and mild reaction conditions. The structural diversity of SGs in *S. rebaudiana* results from sequential glycosylation reactions catalyzed by uridine diphosphate (UDP)-dependent glycosyltransferases (UGTs) [6]. Several UGTs have been cloned and functionally characterized: UGT85C2 and UGT74G1 catalyze the initial glucosylation of the steviol aglycone, whereas UGT91D2 and UGT76G1 mediate downstream β-1,2- and β-1,3-glucosylation of the C-13 sophorosyl moiety, respectively [7]. Enzyme-catalyzed bioconversion has enabled the production of minor SGs, such as RE, RD, and RM, from ST or RA through successive reactions mediated by UGTSL2, UGT91D2, EUGT11, and UGT76G1 [8,9,10,11,12,13].

Despite these advances, existing glycosyltransferase-based biosynthetic approaches have primarily focused on improving the yield of RM without addressing the limited aqueous solubility. Poor aqueous solubility can lead to precipitation and uneven sweetness distribution during beverage processing and storage, ultimately hindering the industrial application of RM [14]. As a complementary enzymatic strategy, α-glucosylation has been investigated to improve the physicochemical and taste-related properties of SGs (Table S1). Dextransucrases derived from *Leuconostoc* species [15,16,17] and cyclodextrin glucanotransferase (CGTase) derived from *Alkalihalobacillus oshimensis* [18] have been reported to catalyze the transfer of α-glucosyl residues to SGs, generating novel α-linked derivatives with substantially improved aqueous solubility. Moreover, unlike β-glucosylation, α-glucosylation has also been reported to confer enhanced thermal and acidic stability, retaining substantially greater resistance under strongly acidic conditions (pH ≤ 2) [17,19]. In addition, α-glucosylated SG derivatives provide a smoother sweetness perception with reduced lingering bitterness [20], underscoring the potential of α-glucosylation as an effective enzymatic route. However, the enzymatic production of minor SGs and the α-glucosylation-mediated improvement of SG functionality have largely been explored as separate strategies. To date, α- and β-glucosylation have not been applied to generate structurally novel RM-like glycosides. Based on the complementary functions reported for α- and β-glucosylation, we hypothesized that their sequential combination could generate novel RM analogs with enhanced aqueous solubility without compromising physicochemical stability or favorable taste-related profiles.

In this study, we established a combinatorial biocatalytic platform for producing novel RM analogs from ST by integrating *L*. *kimchii*-derived dextransucrase-mediated α-glucosylation with UGT76G1-mediated β-glucosylation. We structurally characterized the resulting novel RM analogs, designated RMLC1 and RMLC2, and evaluated their aqueous solubility, thermal and pH stability, in vitro digestibility, and sweetness profiles to assess their potential as next-generation natural sweeteners.

## 2. Materials and Methods

### 2.1. Materials

ST and RA were obtained from MSC Co., Ltd. (Yangsan, Republic of Korea) and Daepyung Co., Ltd. (Seongnam, Republic of Korea), respectively, and RD and RM were purchased from Sigma-Aldrich (St. Louis, MO, USA). The purity of all steviol glycosides was ≥95%. High-performance liquid chromatography (HPLC)-grade water and acetonitrile (MeCN) were purchased from Duksan General Science Co., Ltd. (Seoul, Republic of Korea). Sucrose was obtained from Bio Basic (Markham, ON, Canada), de Man, Rogosa, and Sharpe (MRS) broth from Difco BD (Sparks, MD, USA), and UDP and rat intestinal acetone powder (RIAP) from Sigma-Aldrich. All other chemicals and solvents were of analytical grade and used without further purification.

### 2.2. Preparation of Dextransucrase and UGT76G1

Dextransucrase was prepared as a cell-free crude enzyme from the culture supernatant of *L. kimchii*. Briefly, 100 µL of the bacterial stock was inoculated into 10 mL of MRS broth supplemented with 0.5% (*w*/*v*) sucrose and incubated at 30 °C with shaking at 180 rpm for 24 h. After incubation, the culture broth was centrifuged at 9870× *g* for 20 min, and the supernatant was used as the crude dextransucrase source without further purification.

The UGT76G1 gene from *S. rebaudiana* was amplified by polymerase chain reaction (PCR) using the following primers: forward, 5′-CATCCAAAAAAAAAGTAAGAATTGATGGAAAACAAGACGGAAAC-3′; reverse, 5′-TAATTACATGATATCGACAATCACAAGGAAGAGATGTAGGATACCAAAGA-3′. The amplified fragment was assembled into the linearized pRS426 vector between the GAL10 promoter and the CYC1 terminator using the HiFi DNA Assembly Master Mix (New England Biolabs, Ipswich, MA, USA). The recombinant plasmid pRS426–UGT76G1 was transformed into *S. cerevisiae* CEN.PK2-1C (American Type Culture Collection, Manassas, VA, USA) by the lithium acetate method. A single transformant colony was inoculated into 3 mL of SC–Ura medium and cultured for 24 h at 30 °C, followed by transfer to 25 mL of fresh SC–Ura medium for an additional 24 h. An equal volume of YPG medium was then added, and the culture was incubated for 24 h to induce enzyme expression. Cells were harvested by centrifugation (7010× *g*, 4 °C, 10 min) and lyophilized.

### 2.3. Dextransucrase Activity Assay

Dextransucrase activity was assayed in 20 mM sodium acetate buffer (pH 5.5) containing 200 mM sucrose, using the cell-free supernatant as the enzyme source. The reaction mixture was incubated at 30 °C for 30 min, followed by heat inactivation at 100 °C for 5 min. Fructose released during sucrose conversion was quantified using a d-Glucose/Fructose Assay Kit (Megazyme, Bray, Ireland), and absorbance was measured at 340 nm. One unit (U) of dextransucrase activity was defined as the amount of enzyme required to release 1 μmol of fructose per min under the assay conditions.

### 2.4. Glucosyltransferase Activity Assay

UGT76G1 activity was determined by measuring the consumption of Stev-G 3.1 (designated as RD2 in this study), which was produced by dextransucrase. The 1 mL reaction mixture contained 20 mM sodium phosphate buffer (pH 7.2), 10 mM RD2, 20 mM sucrose, 0.3 mM UDP, 5 mM ethylenediaminetetraacetic acid (EDTA), and 100 mg freeze-dried enzyme. After 30 min of reaction at 50 °C, the reaction was terminated at 100 °C for 5 min and then analyzed using HPLC. One unit (U) of UGT76G1 activity was defined as the amount of enzyme required to convert 1 μmol of RD2 into RMLC1 per min.

### 2.5. Enzymatic Synthesis and Analysis of Novel Glucosyl STs

For RD2 synthesis, the transglucosylation reaction was performed in 20 mM sodium acetate buffer (pH 5.5) containing 40 mM sucrose as the donor, 20 mM ST as the acceptor, and 0.56 U/mL dextransucrase. The reaction mixture was incubated at 30 °C for 24 h, followed by heat inactivation at 100 °C for 5 min. Novel RM analogs, RMLC1 and RMLC2, were synthesized using purified RD2 as the acceptor. The reaction was carried out in 20 mM sodium phosphate buffer (pH 7.2) containing 40 mM sucrose, 20 mM RD2, 0.3 mM UDP, and 5 mM EDTA, and 1.5 U/mL UGT76G1. The reaction mixture was incubated at 50 °C for 12 h, and inactivated at 100 °C for 5 min.

Chromatographic separation for quantitative analysis was achieved using a Shimadzu HPLC system (Shimadzu, Tokyo, Japan) equipped with an SPD-40 UV-Vis detector, LC-40D pumps, an SIL-40 autosampler, and a CTO-40C column oven. The mobile phase consisted of A 10 μL aliquot of each sample was analyzed on a ZORBAX 300SB-C_18_ column (5 μm, 4.6 mm × 150 mm; Agilent Technologies, Palo Alto, CA, USA) using a linear gradient of 0 to 50% MeCN for 35 min at a flow rate of 1.0 mL/min, with detection at 210 nm.

### 2.6. Isolation of RD2 and Novel RM Analogs

The target compounds were purified by the addition of pre-chilled ethanol to the reaction mixture at −80 °C for 2 h. Dextran was removed by centrifugation at 9870× *g* for 20 min, and the resulting supernatant was collected and concentrated using a rotary vacuum evaporator (Eyela, Tokyo, Japan). The concentrated sample was first loaded onto an AW-90 ion-exchange resin column (2.5 cm × 70 cm; Samyang Co., Ltd., Seongnam, Republic of Korea) for deionization. The eluate was subsequently applied to a Diaion HP-20 column (2.5 cm × 70 cm; Samyang Co., Ltd.). After washing the column with 10 column volumes (CV) of water to remove highly polar impurities, the target compounds were eluted with 10 CV of 95% ethanol. The collected eluate was concentrated and further purified by octadecylsilane-modified medium-pressure liquid chromatography (ODS–MPLC; Biotage, Uppsala, Sweden) equipped with an Sfär Bio C18 column (300 Å, 20 µm, 240 g; Biotage). Linear gradient elution was performed, starting with 100% (*v*/*v*) water and gradually increasing to 100% (*v*/*v*) MeCN at a flow rate of 20 mL/min and wavelength of 210 nm.

### 2.7. Structural Elucidation of Novel RM Analogs

Chromatographic separation was performed on an ACQUITY UPLC system (Waters, Milford, MA, USA) with an ACQUITY UPLC HSS T3 column (1.8 μm, 2.1 mm × 50 mm). Gradient elution with solvent A (0.1% formic acid in water) and solvent B (0.1% formic acid in MeCN) was employed at a flow rate of 0.35 mL/min, with the following gradient program: 0–2 min, 2% B; 2–7 min, 2–35% B; 7–13 min, 35–80% B; and 13–16.5 min, 80–100% B. The column oven was kept at 40 °C, and the injection volume was 1 µL. Mass spectrometric detection was conducted using a Xevo G2-XS Q-TOF mass spectrometer (Waters) with an electrospray ionization (ESI) source in positive and negative ion modes. The MS parameters were set as follows: capillary voltage, 2.5 kV; cone voltage, 40 V; cone gas flow, 50 L/h; desolvation gas flow, 600 L/h; desolvation temperature, 200 °C; and collision energy, 6 eV (low) and 25–50 eV (high).

To elucidate the structures of the novel RM analogs, ^1^H and ^13^C-nuclear magnetic resonance (NMR) spectra were recorded on a Varian Unity INOVA 500 and 600 spectrometers (Varian, Palo Alto, CA, USA) at the Gwangju Center of the Korea Basic Science Institute. Approximately 20 mg of each sample was dissolved in 1 mL of pyridine-*d*_5_ and transferred to 5 mm NMR tubes. The connections between the steviol and sugar moieties were further determined by 2D-NMR analyses, including homonuclear correlation spectroscopy (COSY), heteronuclear multiple bond correlation (HMBC), heteronuclear single quantum coherence (HSQC), total correlation spectroscopy (TOCSY), and HSQC–TOCSY.

### 2.8. Evaluation of the Stability of Novel RM Analogs

#### 2.8.1. Thermal and pH Stability

Thermal stability was determined according to the method of Kang et al. [17] with slight modifications. ST, RM, RMLC1, and RMLC2 were dissolved in DMSO/water (10:90, *v*/*v*) to a final concentration of 5 mg/mL. The samples were incubated at 40 °C, 80 °C, and 120 °C for 2 h and analyzed by HPLC.

For pH stability, each compound (5 mg) was incubated in Britton–Robinson (BR) buffer adjusted to pH 2.0 to 10.0 at 80 °C for 2 h under accelerated degradation conditions. The remaining amount of each compound was quantified by HPLC.

#### 2.8.2. Long-Term Storage Stability at Room Temperature

The long-term storage stability of ST, RA, RMLC1, and RMLC2 was evaluated in aqueous solutions prepared at concentrations of 0.1–2% (*w*/*v*). RM was excluded because its low solubility prevented the preparation of a stable aqueous solution. The samples were stored at 25 °C for 30 days, and aliquots were collected on days 0, 7, 15, and 30 and analyzed by HPLC to quantify the residual amount of each compound.

#### 2.8.3. Storage Stability in a Commercial Beverage

To compare the storage stability of ST, RM, RMLC1, and RMLC2 in a commercial beverage, a commercial low-pH beverage (Coca-Cola; The Coca-Cola Company, Atlanta, GA, USA) was used and the stability of each compound was assessed following the method of Wölwer-Rieck et al. [21]. Each compound was prepared in degassed Coca-Cola at a final concentration of 0.08% (*w*/*v*). The samples were incubated at 4 °C, 25 °C, 40 °C, 60 °C, and 80 °C for up to 48 h, and aliquots were analyzed by HPLC.

### 2.9. Physical Characterization of Novel RM Analogs

The physical properties of RM analogs were characterized by Fourier transform infrared spectroscopy (FT-IR), X-ray diffraction (XRD), differential scanning calorimetry (DSC), and thermogravimetric analysis (TGA). FT-IR spectra were recorded in attenuated total reflection (ATR) mode over a wavenumber range of 650–4000 cm^−1^ to examine the functional group profiles, using a Spectrum 3 spectrophotometer (PerkinElmer, Waltham, MA, USA). The crystalline nature of each analog was investigated by XRD using an Empyrean diffractometer (Malvern Panalytical, Malvern, UK) over a 2θ range of 5–60° at a scanning rate of 1°/min. Thermal transition behavior was monitored by DSC over a temperature range of 25–300 °C at a heating rate of 10 °C/min under a nitrogen atmosphere using a DSC3 instrument (Mettler Toledo, Zurich, Switzerland). Thermal degradation profiles were obtained by TGA from 30 to 600 °C at a heating rate of 10 °C/min under nitrogen flow using a TGA2 analyzer (Mettler Toledo).

### 2.10. Determination of Aqueous Solubility

The aqueous solubility of glucosyl STs was determined according to the method of Jozwiakowski and Connors [22], with slight modifications. Briefly, approximately 50–100 mg of each compound was added to 1 mL of deionized water to obtain a supersaturated suspension. After stirring at 25 °C for 24 h, the mixtures were centrifuged at 10,000× *g* for 20 min, and the supernatants were lyophilized and weighed to determine the dissolved amount of each compound.

### 2.11. Determination of Glucose Generation Rate (GGR)

RIAP (4 g) was suspended in 40 mL of 100 mM sodium phosphate buffer (pH 6.0) containing 6.7 mM NaCl and 0.2% sodium azide. The suspension was incubated at 4 °C for 24 h and then centrifuged at 9870× *g* for 10 min. The supernatant was filtered through a 1.5-µm nylon filter (Thermo Fisher Scientific, Waltham, MA, USA), and the filtrate was used as a source of mammalian α-glucosidase.

Substrate solutions (enzymatically modified stevia [Enzyme ST], ST, RA, RD, RM, RD2, RMLC1, and RMLC2) were prepared at a concentration of 1% (*w*/*v*) in 100 mM sodium phosphate buffer (pH 6.0). For each reaction, 100 µL of the substrate solution was mixed with 900 µL of the RIAP extract, and the mixture was incubated at 37 °C with shaking at 180 rpm for 2 h. The enzymatic reactions were terminated by heating at 100 °C for 5 min. The amount of glucose released was quantified at 510 nm using a d-Glucose/Fructose Assay Kit (Megazyme), and time-dependent changes in the substrates were analyzed using HPLC and LC–MS.

### 2.12. Electronic Tongue Analysis

The taste profiles of ST, RA, RM, RMLC1, and RMLC2 were evaluated using an α-Astree II electronic tongue system (Alpha MOS, Toulouse, France) equipped with seven chemical sensors: AHS (sourness), NMS (umami), CTS (saltiness), SCS (bitterness), ANS (sweetness), and CPS and PKS (comprehensive indices), along with an Ag/AgCl reference electrode [23]. Samples were prepared in deionized water at a final concentration of 20 µM, and 25 mL of each sample was used for analysis. Sensor signals were acquired for 150 s at 1 s intervals, followed by a 120 s rinse cycle between successive measurements to minimize sensor carry-over. The stable response region from 130 to 150 s, which exhibited the lowest temporal variation, was used for data analysis. Data acquisition and processing were performed using AlphaSoft 14.1 software (Alpha MOS).

### 2.13. Statistical Analysis

All experiments were conducted in triplicate, and the data are presented as mean ± standard deviation. Statistical analysis was performed using one-way analysis of variance (ANOVA) followed by Duncan’s multiple-range test with GraphPad Prism (version 9.3.1; GraphPad Software, San Diego, CA, USA). Differences were considered statistically significant if the *p*-value was less than 0.05.

## 3. Results and Discussion

### 3.1. Production and Isolation of Novel RM Analogs

The glucosylation of ST was catalyzed by *L. kimchii* dextransucrase using sucrose as the donor substrate, yielding RD2 as the primary product. HPLC analysis confirmed the conversion of ST to RD2 (Figure S1). After purification by Diaion HP-20 resin chromatography and MPLC, 32.2 mg of RD2 was obtained from 161 mg of ST, corresponding to an isolated mass recovery of 20.0%.

**Figure 1:**
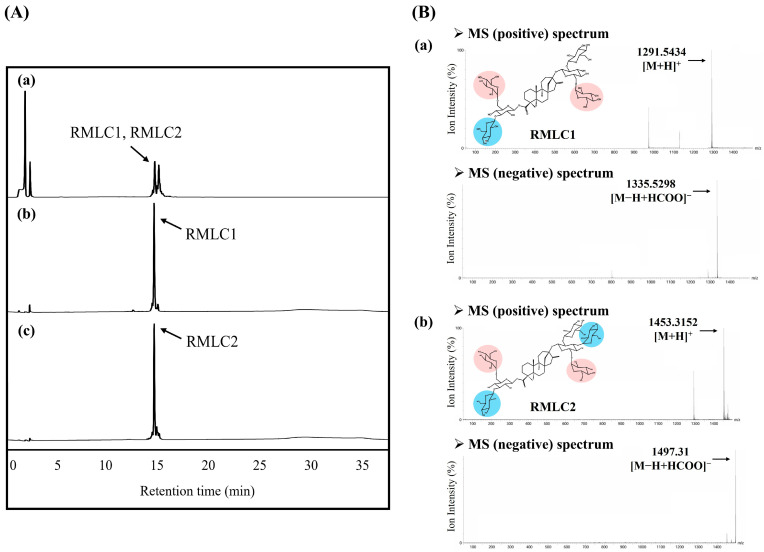
Production and isolation of novel RM analogs. (**A**) HPLC chromatograms: (**a**) RMLC1 and RMLC2 in the enzymatic reaction mixture; (**b**) the isolated RMLC1; and (**c**) the isolated RMLC2. (**B**) LC-ESI/MS spectra: (**a**) the isolated RMLC1 and (**b**) the isolated RMLC2. Abbreviations: RMLC1: rebaudioside M analog 1, RMLC2: rebaudioside M analog 2. Pink indicates α-glucosylation, whereas blue indicates β-glucosylation.

RD2 subsequently served as the acceptor substrate for further glucosylation by recombinant UGT76G1. The enzyme efficiently catalyzed the production of two novel RM analogs, RMLC1 and RMLC2, with RD2 substrate conversion exceeding 95% (Figure 1A). From 226 mg of RD2, 100 mg of RMLC1 and 86.2 mg of RMLC2 were isolated, corresponding to isolated mass recoveries of 44.3% and 38.1%, respectively. All isolated compounds exhibited chromatographic purity exceeding 95%.

### 3.2. Structural Elucidation of Novel RM Analogs

The structures of the isolated RM analogs were elucidated using LC-ESI/MS, 1D-NMR (^1^H, ^13^C), and 2D-NMR (^1^H–^1^H COSY, HMBC, HSQC, TOCSY, and HSQC-TOCSY) analyses. The NMR data of RD2 were in excellent agreement with those previously reported for *O*-α-d-glucosyl-(1″→6′)-*O*-α-d-glucosyl-(1′′′′′→6‴)-stevioside [17]. Accordingly, the complete spectral assignments of RD2 are provided in the Supplementary Materials (Figures S2–S9; Table S2), and only the characterization of the two novel analogs is detailed below.

**Figure 2:**
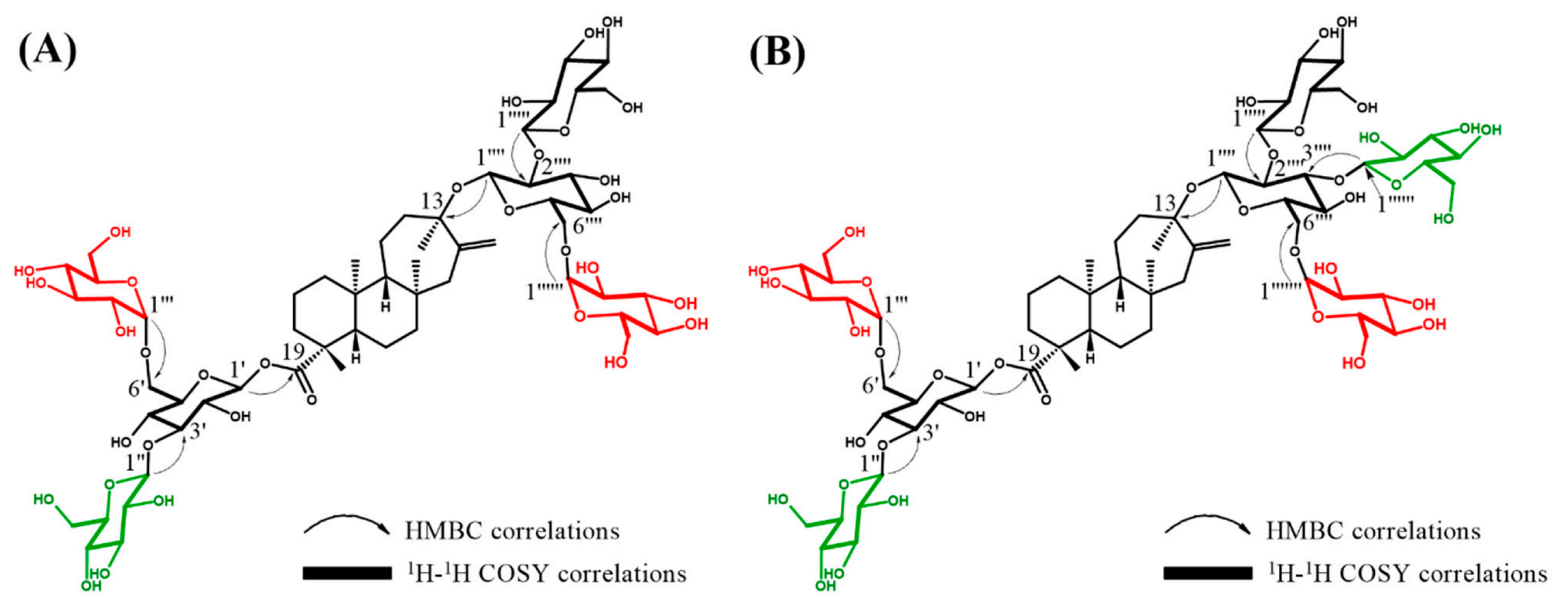
Structures of (**A**) RMLC1, *O-*β-d-glucosyl-(1′′→3′)-*O-*α-d-glucosyl-(1′′′→6′)-*O-*α-d-glucosyl-(1′′′′′′→6′′′′)-stevioside and (**B**) RMLC2, *O-*β-d-glucosyl-(1′′→3′)-*O-*α-d-glucosyl-(1′′′→6′)-*O-*β-d-glucosyl-(1′′′′′′→3′′′′)-*O-*α-d-glucosyl-(1′′′′′′′→6′′′′)-stevioside. Red indicates α-glucosylation, whereas green indicates β-glucosylation.

The ESI/MS spectrum of RMLC1 in the positive ion mode presented a major peak [M + H]^+^ at *m*/*z* 1291.5434, consistent with the attachment of three glucose moieties to ST (MW: 804.9 g/mol) (Figure 1B). Compared to the ^1^H and ^13^C NMR spectra of RD2, RMLC1 additionally contained a sugar moiety corresponding to proton signals at δ 5.34, 4.03, 4.19, 4.09, 3.95, 4.50, and 4.20 and carbon signals at δ 105.27, 74.20, 78.36, 71.97, 78.71, and 62.77 (Figures S10 and S11; Table S3). The coupling constant (*J* = 8.0 Hz) of the anomeric proton signal at δ 5.34 in the ^1^H NMR spectrum indicated that the additional sugar moiety was β-d-glucose, as further supported by ^1^H-^1^H COSY and TOCSY data (Figures S12 and S13). In the HMBC spectrum, the correlation between δ 5.34 (H-1″) and 89.07 (C-3′) was observed, indicating that C-1″ of β-d-glucose was etherified with C-3′ of RD2 (Figure S14). Therefore, RMLC1 was identified as *O*-β-d-glucosyl-(1″→3′)-*O*-α-d-glucosyl-(1‴→6′)-*O*-α-d-glucosyl-(1′′′′′′→6′′′′)-stevioside (Figure 2A).

The ESI/MS spectrum of RMLC2 exhibited a protonated molecular ion peak [M + H]+ at *m*/*z* 1453.3152 due to the addition of four glucose units to ST (Figure 1B). Compared with RMLC1, one additional β-d-glucose moiety corresponding to proton signals at δ 5.32, 4.12, 4.07, 4.27, 4.24, 4.54, and 4.20 and carbon signals at δ 104.87, 74.36, 78.71, 72.23, 78.21, and 62.71 was newly observed in RMLC2 (Figures S16–S21; Table S4). The anomeric proton at δ 5.32 exhibited a coupling constant of *J* = 8.0 Hz, confirming its β-configuration. The HMBCs between δ 5.32 (H-1′′′′′′′) and δ 87.72 (C-3′′′′) established that newly added β-d-glucose was linked to the C-3′′′′ of RMLC1. Thus, RMLC2 was elucidated as *O*-β-d-glucosyl-(1″→3′)-*O*-α-d-glucosyl-(1‴→6′)-*O*-β-d-glucosyl-(1′′′′′′→3′′′′)-*O*-α-d-glucosyl-(1′′′′′′′→6′′′′)-stevioside (Figure 2B). Based on these structurally confirmed linkage patterns, the regioselectivity of the sequential reaction likely reflects enzyme-specific substrate orientation and steric accessibility. Dextransucrase appears to favor the accessible primary C-6 hydroxyl groups, whereas UGT76G1 preferentially positions the relevant C-3 hydroxyl group for β-1,3-glucosylation, resulting in the selective formation of RMLC1 and RMLC [24].

### 3.3. Evaluation of Stability Properties of Novel RM Analogs

#### 3.3.1. Thermal and pH Stability

**Figure 3:**
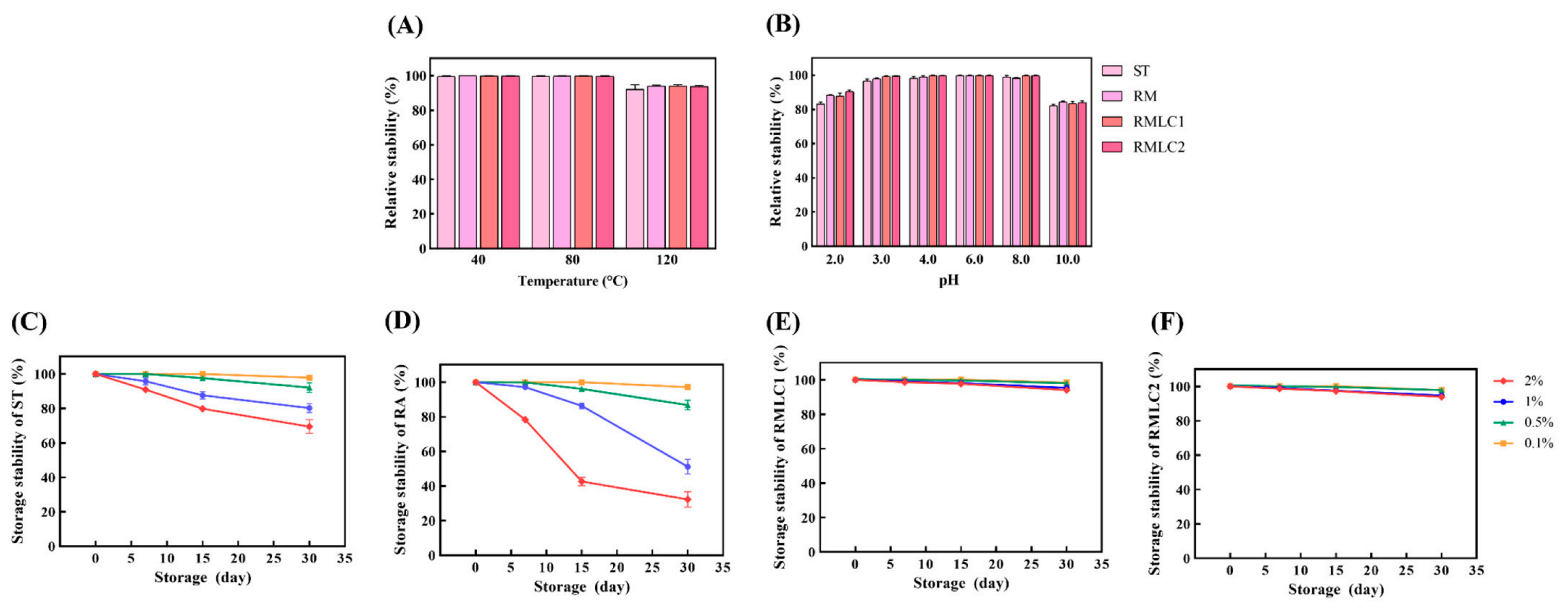
Stability tests of steviol glycosides under different conditions. (**A**) Thermal stability of ST, RM, RMLC1, and RMLC2; (**B**) pH stability of ST, RM, RMLC1, and RMLC2; (**C**) storage stability of ST; (**D**) storage stability of RA; (**E**) storage stability of RMLC1; (**F**) storage stability of RMLC2. Abbreviation: ST: stevioside, RA: rebaudioside A, RM: rebaudioside M, RMLC1: rebaudioside M analog 1, RMLC2: rebaudioside M analog 2.

The suitability of novel RM analogs as potential sweeteners was evaluated for thermal and pH stability. When heated at 40 °C and 80 °C, all compounds (ST, RM, RMLC1, and RMLC2) showed no significant thermal decomposition (Figure 3A). Even at 120 °C, each compound retained more than 92% of its initial concentration (ST, 92.01%; RM, 94.07%; RMLC1, 94.08%; RMLC2, 93.74%). This high thermostability is consistent with the intrinsic resistance of the steviol aglycone backbone to thermal degradation [25]. It indicates that the additional glucosylation in RMLC1 and RMLC2 does not compromise thermal stability, supporting their applicability in high-temperature food processing such as baking and pasteurization [26].

pH stability was evaluated at 80 °C across pH values of 2.0–10.0 (Figure 3B). Between pH 3.0 and 8.0, ST, RM, RMLC1, and RMLC2 maintained more than 96% of their initial concentrations. At pH 3.0–10.0, the stability of RMLC1 and RMLC2 bearing both α- and β-glycosidic linkages was comparable to that of ST and RM containing only β-glycosidic linkages (*p* > 0.05). Under strongly acidic conditions (pH 2.0), however, RM (88.24%), RMLC1 (87.70%), and RMLC2 (89.86%) retained significantly higher concentrations than ST (83.22%) (*p* < 0.05). This observation is in agreement with Son et al. [27], who reported that α-glucosylated ST derivatives exhibited enhanced acid stability at pH 2.0 compared to unmodified ST. Although the precise mechanism underlying this enhanced acid resistance has not been fully elucidated, longer and more branched sugar chains at C-13 may sterically hinder the access of solvent molecules to the acid-labile C-19 ester bond [28,29]. No significant differences were observed among RM, RMLC1, and RMLC2 (*p* > 0.05), indicating that the UGT76G1-catalyzed β-(1→3) glucosylation did not attenuate the acid stability, which remained equivalent to that of authentic RM.

SGs are known to be stable in solution at pH 4.0–8.0, with their stability decreasing under extreme pH and elevated temperatures [13,30,31,32,33]. Since RA is the most widely adopted steviol glycoside sweetener in the food industry, RM has been reported to behave similarly to RA under typical food-processing conditions, with its stability decreasing at elevated temperatures [15,34,35]. Given this RA-like stability profile of RM, the demonstrated thermal and pH stability of RMLC1 and RMLC2 further support their practical applicability as alternatives to conventional ST and RA in acidic and high-temperature food processing.

#### 3.3.2. Long-Term Storage Stability at Room Temperature

While the long-term stability of RM in the dry state at ambient temperature has been documented (~1% loss over 12 weeks at 25 °C), comparable data under aqueous conditions are limited [4]. Hence, in this study, the storage stability of ST, RA, RMLC1, and RMLC2 in aqueous solution was assessed at 25 °C for up to 30 days. All 0.1% and 0.5% (*w*/*v*) samples retained more than 92% of their initial concentrations over 30 days, except for 0.5% (*w*/*v*) RA. However, the stability of ST decreased to 80.12% at 1% (*w*/*v*) and 69.40% at 2% (*w*/*v*), with visible precipitation noted at higher concentrations, likely attributable to supersaturation exceeding its limited aqueous solubility (Figure 3C). RA showed a similar but more pronounced tendency, with sharp declines to 51.20% and 32.33% noted at 1% and 2% (*w*/*v*), respectively (Figure 3D), consistent with its known low water solubility (~0.13% (*w*/*v*) at 25 °C) [36]. In contrast, RMLC1 and RMLC2 were highly stable across the entire concentration range of 0.1–2% (*w*/*v*). Even at 2% (*w*/*v*), 94.06% and 93.93% of the initial concentrations of RMLC1 and RMLC2 were retained after 30 days (Figure 3E,F), respectively, indicating significantly superior stability compared to ST and RA.

The superior long-term stability of RMLC1 and RMLC2 at higher concentrations can be attributed to two complementary factors: (i) the α-glucosylation at both the C-13 and C-19 positions increases the overall hydrophilicity of the steviol backbone, thereby enhancing aqueous solubility and preventing supersaturation-driven precipitation [17,19]; and (ii) these additional sugar moieties provide steric protection of the acid-labile C-19 ester bond, reducing hydrolytic degradation during prolonged storage, consistent with the mechanism proposed for RM [4]. Together with their demonstrated thermal and pH stability, these results support the potential application of RMLC1 and RMLC2 in water-based formulations such as ready-to-drink beverages [37]. However, extended storage studies are needed to fully validate their shelf-life performance under commercial conditions.

#### 3.3.3. Storage Stability in a Commercial Beverage

The stability of SGs in beverage systems is affected by both formulation and processing variables, including pH, acidulant type, buffer capacity, and ionic strength [29,38,39]. Accordingly, a commercial low-pH beverage (Coca-Cola) was selected as a model beverage matrix, and the storage stability of ST, RM, RMLC1, and RMLC2 was evaluated under accelerated thermal storage conditions (up to 80 °C for 48 h; Table 1). All four compounds remained intact at 4 °C and 25 °C. However, after 48 h at 80 °C, ST and RM degraded extensively, retaining only 4.95% and 4.46% of their initial concentrations, respectively, whereas RMLC1 and RMLC2 retained 14.38% and 14.68%, respectively, corresponding to approximately 3-fold higher retention than RM.

RMLC1 contains the same total number of glucose residues as RM (six per molecule), but differs in that two β-glycosidic linkages are replaced by α-glycosidic linkages. The greater stability of RMLC1 than RM indicates that the introduced α-glycosidic linkages contributed to resistance against acid-thermal degradation. The comparable stability of RMLC1 and RMLC2, despite the additional β-glucose unit in RMLC2, further suggests that the additional β-glucosyl residue did not confer a measurable benefit in stability under the conditions tested. These results indicate that the introduction of α-glycosidic linkages enhanced the resistance of RMLC1 and RMLC2 to acid-thermal hydrolysis, consistent with previous reports demonstrating greater stability of α-glycosylated SGs in coke and orange juice under high-temperature conditions [17,19]. Nevertheless, further studies using model beverage systems are warranted to elucidate the mechanisms underlying the observed stability differences and to assess the applicability of these compounds in more complex food matrices.

Overall, RMLC1 and RMLC2 demonstrated greater acid–thermal stability than ST and RM in the commercial low-pH cola matrix, suggesting their potential for application in thermally processed low-pH beverages.

### 3.4. Physical Characterization of Novel RM Analogs

FT–IR, XRD, DSC, and TGA analyses were performed to elucidate the structural and thermal properties of RM and novel RM analogs, RMLC1 and RMLC2.

**Figure 4:**
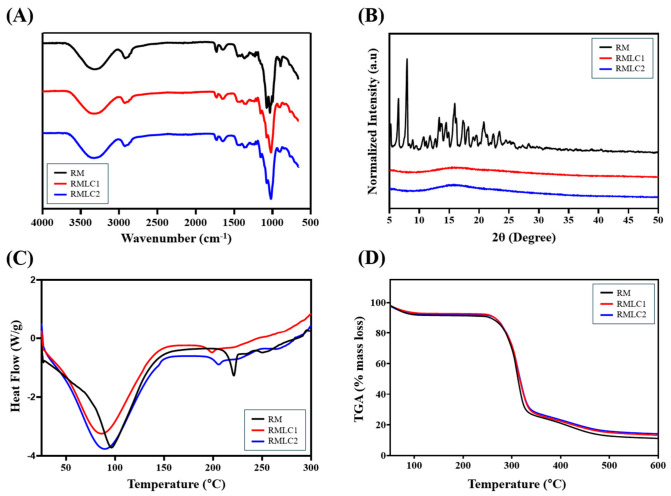
Physical characterization of RM, RMLC1, and RMLC2. (**A**) Fourier-transform infrared spectroscopy spectra (FT-IR); (**B**) X-ray diffraction pattern (XRD); (**C**) differential scanning calorimetry thermogram (DSC); (**D**) thermogravimetric analysis (TGA). Abbreviations: RM: rebaudioside M, RMLC1: rebaudioside M analog 1, RMLC2: rebaudioside M analog 2.

FT-IR spectral analysis confirmed the successful incorporation of additional glucosyl moieties into the stevioside (Figure 4A). The spectra of RMLC1 and RMLC2 exhibited a broadened O–H stretching band (3000–3600 cm^−1^) and an intensified C–O–C asymmetric stretching signal (~1150 cm^−1^) relative to stevioside, consistent with the formation of additional hydrogen bonds and new α-/β-glycosidic linkages [40]. XRD patterns revealed a distinct structural transition from a crystalline to an amorphous state (Figure 4B). RM displayed sharp diffraction peaks at 5.19°, 6.44°, and 7.87° (FWHM = 0.246°), indicative of a well-ordered crystalline lattice, whereas RMLC1 and RMLC2 exhibited broad amorphous halos (FWHM = 16.82° and 47.64°, respectively). Peak-area deconvolution yielded relative crystallinity indices of 34.4% for RM and below 0.5% for both RMLC1 and RMLC2, with good agreement between the observed and fitted profiles (R^2^ = 0.972–0.998). These results indicate that glucosylation markedly disrupted long-range molecular order, consistent with previous reports that glucan modification, branching, and structural rearrangements weaken molecular packing and enhance aqueous dispersibility [41,42].

Thermal transitions measured by DSC further confirmed the loss of crystalline order in RM analogs (Figure 4C). RM showed a sharp endothermic peak at 221.2 °C (ΔH = −8.68 J/g), whereas RMLC1 and RMLC2 exhibited broader transitions at 198.7 °C (ΔH = −2.44 J/g) and 205.5 °C (ΔH = −2.66 J/g), indicating that glucosylation disrupted intermolecular packing and prevented re-establishment of long-range crystalline order. The higher onset temperature of RMLC2 suggests a relatively more organized local microenvironment within the amorphous matrix. TGA profiles revealed that initial moisture loss up to 183 °C was highest in RM (8.47%), followed by RMLC2 (7.83%) and RMLC1 (7.07%) (Figure 4D). The lowest hygroscopicity of RMLC1 likely reflects the compact axial orientation of α-1,6 linkages, which facilitates intramolecular hydrogen bonding and reduces hydroxyl group availability for water interaction. In RMLC2, additional β-1,3 glucosylation reorients hydroxyl groups toward a more solvent-exposed equatorial configuration, partially counteracting this shielding effect. Collectively, the degree and linkage type of glucosylation act as structural determinants governing thermal stability and hygroscopic behavior across the three compounds.

### 3.5. Determination of Aqueous Solubility

The limited aqueous solubility of SGs such as ST and RM represents a significant challenge for their food industry applications, contributing to uneven dispersion, processing difficulties, and an undesirable rough mouthfeel caused by undissolved particles [43]. Therefore, enhancing solubility is critical not only for improving functional performance as sweeteners but also for enabling effective blending with other sweeteners in complex, multicomponent formulations [44].

The aqueous solubilities of ST, RA, RD, RM, RMLC1, and RMLC2 were determined in this study (Table 2). ST and RA exhibited solubilities of 12.02 and 3.60 mg/mL, respectively. In contrast, RD and RM showed substantially lower solubilities of 0.57 and 1.30 mg/mL, respectively, consistent with the previously reported low aqueous solubility of RM (1 mg/mL at 25 °C), which is attributed to its highly glycosylated yet β-linked sugar moiety [8]. Remarkably, RMLC1 and RMLC2 exhibited significantly higher solubilities of 180.60 and 164.40 mg/mL, respectively, representing increases of approximately 139- and 127-fold relative to RM. The pronounced solubility enhancement observed in RMLC1 and RMLC2 is attributable to the introduction of α-glycosidic linkages. In contrast to α-linkages, β-glycosidic linkages tend to stabilize intramolecular hydrogen bonds between ring oxygen atoms and adjacent hydroxyl groups, suppressing conformational fluctuation upon hydration and reducing the probability of the excluded-volume effect of water molecules, collectively lowering aqueous solubility through conformational and water-entropy penalties [45]. This linkage-dependent solubility difference is well demonstrated by the disaccharide pair maltose (α-1,4 linkage) and cellobiose (β-1,4 linkage), in which maltose exhibits significantly greater aqueous solubility owing to the same entropic mechanisms [46,47]. A well-established precedent for α-glucosylation as a strategy to enhance solubility exists in the case of hesperidin, a flavonoid abundant in citrus peel, which exhibits exceedingly low water solubility due to its β-glycosidic structure, causing precipitation in beverages. Enzymatic α-glucosylation has been shown to increase its solubility by more than 10,000-fold relative to the native form [48]. Thus, the improved aqueous solubility of RMLC1 and RMLC2 may facilitate more uniform dissolution and sweetness delivery while reducing the likelihood of precipitation or recrystallization during processing and storage, without necessarily increasing their intrinsic sweetness potency.

### 3.6. Determination of GGR

**Figure 5:**
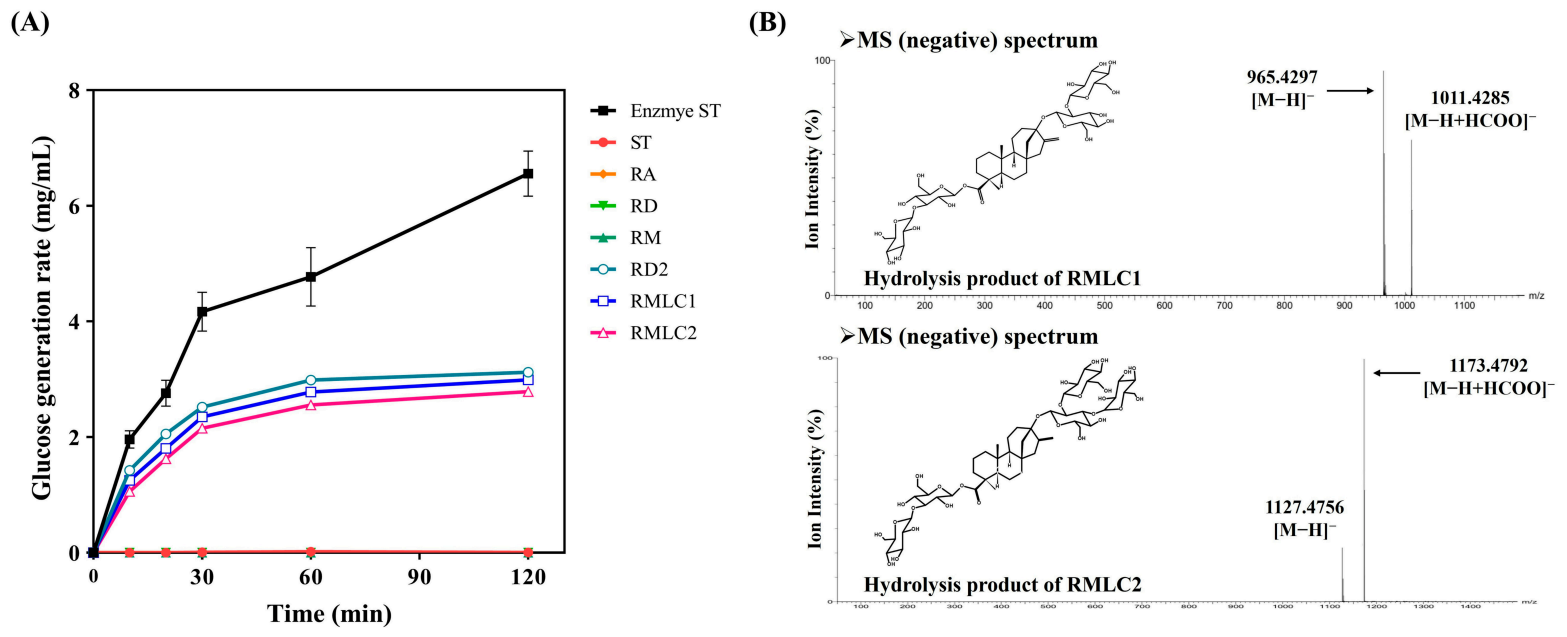
Glucose generation rate (GGR) of steviol glycosides and LC-ESI/MS analysis. (**A**) The GGR of Enzyme ST (■), ST (●), RA (◆), RD (▼), RM (▲), RD2 (○), RMLC1 (□), and RMLC2 (△); (**B**) LC-ESI/MS spectra of enzymatically hydrolyzed RMLC1 (upper) and RMLC2 (lower). Abbreviations: Enzyme ST: enzymatically modified stevia, ST: stevioside, RA: rebaudioside A, RD: rebaudioside D, RM: rebaudioside M, RD2: stev-G 3.1, RMLC1: rebaudioside M analog 1, RMLC2: rebaudioside M analog 2.

A critical consideration for novel sweeteners is their susceptibility to intestinal digestion, particularly given that α-glucosyl moieties were introduced in RMLC1 and RMLC2 to enhance solubility. To evaluate this, digestibility was assessed using a commercial RIAP, which contains the key α-glucosidase complexes maltase–glucoamylase and sucrase–isomaltase, responsible for carbohydrate hydrolysis in the small intestine [49,50,51]. These enzymes exhibit exo-hydrolytic activity toward α-1,2-, α-1,4-, and α-1,6-glycosidic linkages [52]. As expected, β-glucosylated SGs (ST, RA, RD, and RM) were resistant to enzymatic hydrolysis with negligible glucose release, confirming strict enzyme specificity for α-glycosidic linkages. In contrast, the positive control EMS, which contains diverse α-glycosidic bonds (α-1,3, α-1,4, and α-1,6), was readily hydrolyzed, releasing 6.89 mg of glucose. Notably, RMLC1 and RMLC2 released only 2.99 and 2.79 mg of glucose, respectively, less than half the amount generated from EMS (Figure 5A), demonstrating substantial resistance to intestinal digestion despite harboring α-glycosidic linkages. From a nutritional perspective, the limited hydrolysis indicates minimal contribution to postprandial glucose levels, supporting their classification as low-calorie sweeteners. Nevertheless, the RIAP-based assay represents a simplified in vitro model and does not fully reproduce the sequential complexity of human gastrointestinal digestion. Further in vivo studies are therefore warranted to characterize the gastrointestinal digestibility of RMLC1 and RMLC2.

To further elucidate the degradation pathway, LC–MS analysis of the digestion products was performed. Hydrolysis was found to occur selectively at the α-linkages of RMLC1 and RMLC2, yielding rebaudioside E (RE) and RD-like structures as the main products (Figure 5B). This metabolic behavior parallels that of conventional SGs, which undergo stepwise deglycosylation to steviol by colonic microbiota, followed by hepatic conjugation to steviol glucuronide for urinary excretion [53,54,55,56]. This predictable and well-characterized metabolic pathway provides a strong foundation for the safety assessment of these novel RM analogs. 

### 3.7. Electronic Tongue Analysis

**Figure 6:**
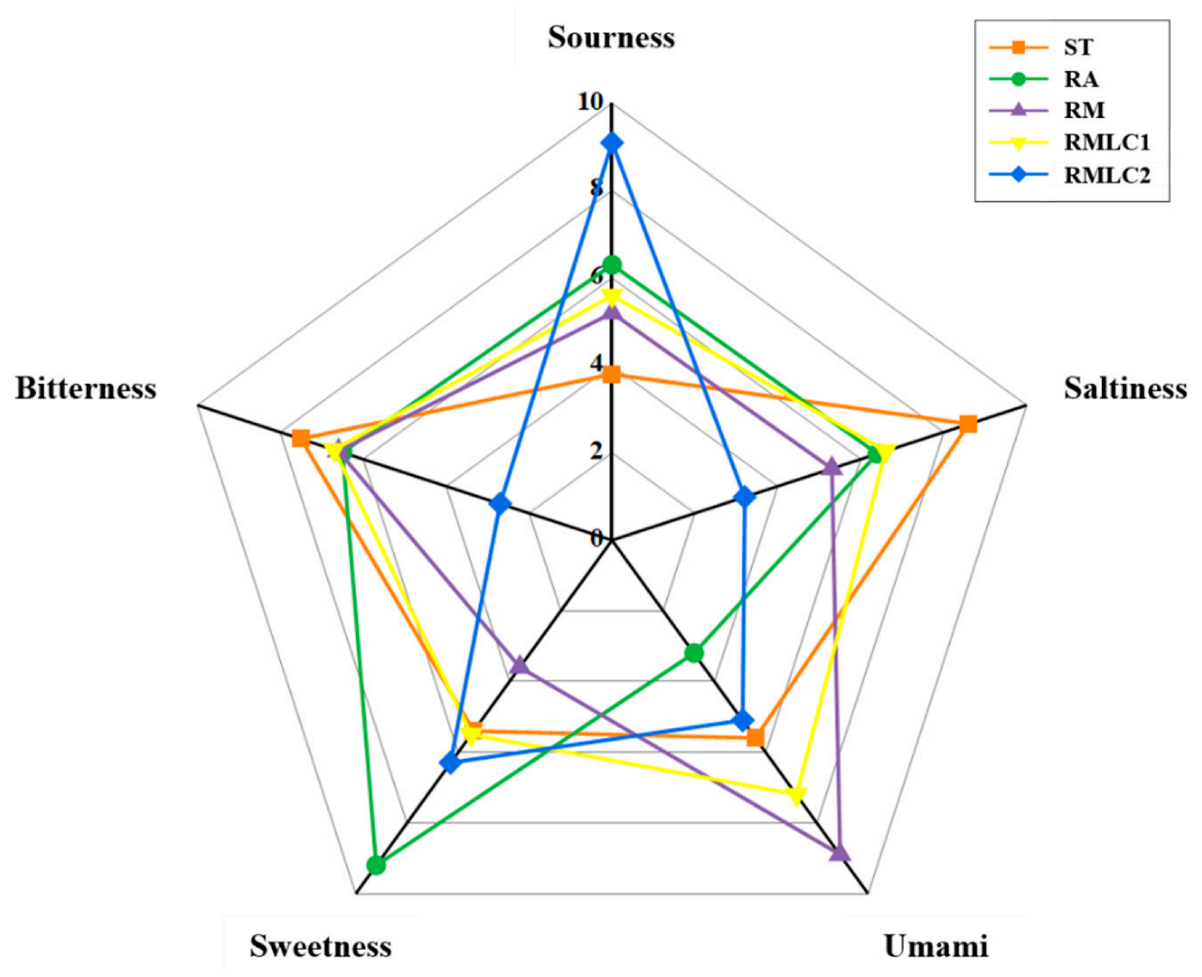
Taste evaluation of steviol glycosides by electronic tongue. Abbreviations: ST: stevioside, RA: rebaudioside A, RM: rebaudioside M, RMLC1: rebaudioside M analog 1, RMLC2: rebaudioside M analog 2.

An electronic tongue was used to compare the sensory profiles of five SGs: ST, RA, RM, RMLC1, and RMLC2. Figure 6 presents a radar plot of the five potentiometric channels (AHS, CTS, NMS, ANS, and SCS). Due to known cross-selectivity among sensors, interpretation was based on overall multichannel patterns rather than one-to-one taste mapping [57].

ST showed the highest CTS value (8.6) among the five SGs, indicating a relatively strong saltiness response. RA exhibited the highest ANS response (9.2) with a moderate SCS signal (6.5), indicating a sweet profile with slight lingering bitterness, consistent with human sensory evaluations of RA [58]. RM showed the highest NMS value (8.9), suggesting a more complex taste profile with umami-like contributions, while prior reports indicate that RM delivers a comparatively cleaner sweetness than RA in a matrix-dependent manner [3,4]. In contrast, RMLC1 produced a relatively balanced pattern without dominant responses on any individual sensor channel, suggesting high blending versatility. RMLC2 displayed the most sweetness-forward profile, combining the highest AHS value (9.1) with the lowest SCS value (2.7), indicative of an enhanced sweetness signal with minimal bitterness/astringency response. The cleaner profiles of the α-glucosylated analogs agree with previous findings that glucansucrase-mediated α-glucosylation or related transglycosylation can reduce metallic or lingering off-notes and improve palatability relative to the parent glycosides [59,60,61]. Therefore, while RMLC1 may offer a more balanced taste profile suitable for blending applications, RMLC2 appears particularly suited as a sweetness booster with minimal bitterness. However, the electronic tongue cannot fully replace human sensory evaluation, and future studies incorporating sensory evaluation by independent panelists are needed to provide more comprehensive sensory characterization.

## 4. Conclusions

The broader application of SGs as high-intensity sweeteners remains constrained by limitations in aqueous solubility, storage stability, and undesirable taste attributes. In this study, a dual-enzyme system comprising *L. kimchii* dextransucrase and *S. rebaudiana* UGT76G1 was established to synthesize novel RM analogs, RMLC1 and RMLC2, through the controlled introduction of α- and β-glucosyl residues, with overall conversion yields exceeding 95%. Both analogs exhibited high thermostability and pH stability, significantly enhanced storage stability at room temperature in aqueous solution. Under accelerated low-pH beverage conditions, RMLC1 and RMLC2 retained markedly higher residual concentrations than ST and RM, confirming their enhanced acid and heat resistance. In an in vitro digestion assay, both compounds exhibited a slower hydrolysis rate than the α-linked EMS and were converted into RE- or RD-like products, as confirmed by LC–MS, indicating the selective cleavage of α-linkages while preserving the steviol backbone. Electronic tongue profiling further confirmed that RMLC1 exhibited a balanced, blend-friendly profile, whereas RMLC2 exhibited a sweetness-forward profile with minimal bitterness/astringency. Taken together, these results support sequential α/β-glucosylation as a useful strategy for improving the physicochemical, digestive, and taste-related properties of SGs. RMLC1 and RMLC2 are therefore promising next-generation sweeteners in high-temperature manufacturing processes and shelf-stable, low-pH beverage applications, owing to the conformational stabilization conferred by α- and β-glycosidic linkages and their acid resilience relative to ST and RM. However, further studies are warranted to elucidate their in vivo digestibility and glycemic responses, to validate their sensory attributes through trained and consumer panels, and to investigate their functional performance across diverse food and beverage matrices with varying compositions under industrial manufacturing conditions.

## Figures and Tables

**Table 1:** Storage stability of steviol glycosides in a soft drink.

	Temperature (°C)	Time (h)	ST	RM	RMLC1	RMLC2
Coke (pH 2.5)	4	24	100 ^a^	100 ^a^	100 ^a^	100 ^a^
		48	100 ^a^	100 ^ab^	100 ^a^	100 ^a^
	25	24	100.05 ± 0.18 ^a^	99.99 ± 0.77 ^ab^	100.06 ± 0.12 ^a^	100.12 ± 0.35 ^a^
		48	99.99 ± 0.01 ^a^	100.17 ± 0.30 ^ab^	100.08 ± 0.14 ^a^	99.78 ± 0.58 ^a^
	40	24	96.68 ± 0.75 ^b^	99.97 ± 0.79 ^ab^	98.93 ± 0.98 ^a^	100 ± 0.35 ^a^
		48	91.67 ± 1.16 ^c^	98.57 ± 1.26 ^b^	98.94 ± 1.13 ^a^	100 ± 0.60 ^a^
	60	24	76.54 ± 1.22 ^d^	78.35 ± 0.92 ^c^	89.74 ± 1.04 ^b^	90.57 ± 0.96 ^b^
		48	62.93 ± 0.79 ^e^	59.84 ± 0.88 ^d^	74.52 ± 0.86 ^c^	76.72 ± 0.87 ^c^
	80	24	18.56 ± 0.73 ^f^	28.00 ± 1.24 ^e^	41.28 ± 1.09 ^d^	48.42 ± 0.88 ^d^
		48	4.95 ± 0.93 ^g^	4.46 ± 0.55 ^f^	14.38 ± 1.10 ^e^	14.68 ± 0.90 ^e^

The difference in values within the column was analyzed using one-way ANOVA. Duncan’s multiple analysis was used, and different lowercase letters indicate a significant difference (*p* < 0.05). Abbreviations: ST: stevioside, RM: rebaudioside M, RMLC1: rebaudioside M analog 1, RMLC2: rebaudioside M analog 2.

**Table 2:** Aqueous solubility of glucosylated steviol glycosides.

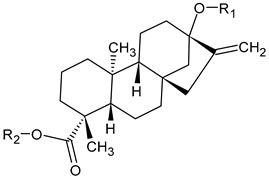					
**Compound**	**R_1_**	**R_2_**	**Molecular Weight**	**Aqueous Solubility** **(mg/mL, 25 °C)**	**Reference**
Stevioside	β-Glc-β-Glc (2→1)	β-Glc	804.87	12.02 ± 0.33	[19]
Rebaudioside A	β-Glc-β-Glc (2→1) -β-Glc (3→1)	β-Glc	967.01	3.60 ± 0.16	[19]
Rebaudioside D	β-Glc-β-Glc (2→1) -β-Glc (3→1)	β-Glc-β-Glc (2→1)	1129.01	0.57 ± 0.05	[19]
Rebaudioside M	β-Glc-β-Glc (2→1) -β-Glc (3→1)	β-Glc-β-Glc (2→1) -β-Glc (3→1)	1291.29	1.3 ± 0.85	[4]
RMLC1	β-Glc-β-Glc (2→1) -α-Glc (6→1)	β-Glc-β-Glc (3→1) -α-Glc (6→1)	1291.29	>180.6	In this study
RMLC2	β-Glc-β-Glc (2→1) -β-Glc (3→1) -α-Glc (6→1)	β-Glc-β-Glc (3→1) -α-Glc (6→1)	1453.29	>164.4	In this study

Abbreviations: RMLC1: rebaudioside M analog 1, RMLC2: rebaudioside M analog 2.

## Data Availability

The original contributions presented in this study are included in the article/Supplementary Materials. Further inquiries can be directed to the corresponding authors.

## Supplementary Materials

The following supporting information can be downloaded at: https://www.mdpi.com/article/10.3390/foods15152647/s1, Figure S1. HPLC chromatograms of (A) ST standard; (B) the reaction mixture consisting of 40 mM sucrose, 20 mM ST, and 0.56 U/mL dextransucrase in 20 mM sodium acetate buffer (pH 5.5) was incubated at 30oC for 48 h; (C) the reaction mixture consisting of 40 mM sucrose, 20 mM ST, and 0.56 U/mL dextransucrase in 20 mM sodium acetate buffer (pH 5.5) was incubated at 30oC for 48 h. Abbreviations: ST: stevioside, RD2: Stev-G 3.1. Figure S2. LC-ESI/MS analysis of the isolated RD2. Figure S3. ^1^H NMR (500 MHz) spectrum of RD2 in pyridin-*d*_5_. Figure S4. ^13^C NMR (125 MHz) spectrum of RD2 in pyridin-*d*_5_. Figure S5. ^1^H-^1^H COSY spectrum of RD2. Figure S6. TOCSY spectrum of RD2. Figure S7. HSQC spectrum of RD2. Figure S8. HMBC spectrum of RD2. Figure S9. Structural analysis of RD2. Figure S10. ^1^H NMR (500 MHz) spectrum of rebaudioside M analog 1 (RMLC1) in pyridin-*d*_5_. Figure S11. ^13^C NMR (125 MHz) spectrum of rebaudioside M analog 1 (RMLC1) in pyridin-*d*_5_. Figure S12. ^1^H-^1^H COSY spectrum of rebaudioside M analog 1 (RMLC1). Figure S13. TOCSY spectrum of rebaudioside M analog 1 (RMLC1). Figure S14. HSQC spectrum of rebaudioside M analog 1 (RMLC1). Figure S15. HMBC spectrum of rebaudioside M analog 1 (RMLC1). Figure S16. ^1^H NMR (500 MHz) spectrum of rebaudioside M analog 2 (RMLC2) in pyridin-*d*_5_. Figure S17. ^13^C NMR (125 MHz) spectrum of rebaudioside M analog 2 (RMLC2) in pyridin-*d*_5_. Figure S18. ^1^H-^1^H COSY spectrum of rebaudioside M analog 2 (RMLC2). Figure S19. TOCSY spectrum of rebaudioside M analog 2 (RMLC2). Figure S20. HSQC spectrum of rebaudioside M analog 2 (RMLC2). Figure S21. HMBC spectrum of rebaudioside M analog 2 (RMLC2). Table S1. Comparison of representative enzymatic glucosylation strategies for steviol glycosides. Table S2. ^1^H- and ^13^C-NMR data of RD2 in pyridine-*d*_5_. Table S3. ^1^H- and ^13^C- NMR data of RMLC1, O-β-D-glucosyl-(1′′→3′)-O-α-D-glucosyl-(1′′′→6′)-O-α-D-glucosyl-(1′′′′′′→6′′′′)-stevioside. Table S4. ^1^H- and ^13^C- NMR data of RMLC2, O-β-D-glucosyl-(1′′→3′)-O-α-D-glucosyl-(1′′′→6′)-O-β-D-glucosyl-(1′′′′′′→3′′′′)-O-α-D-glucosyl-(1′′′′′′′→6′′′′)-stevioside.
