# Supplementary material for: Dual-Enzyme Biocatalytic Synthesis of Novel Rebaudioside M Analogs with Enhanced Aqueous Solubility, Physicochemical Stability, and Sensory Profiles
Source: Foods. 2026 Jul 28;15(15):2647. doi: 10.3390/foods15152647 (PMC13464401; doi:10.3390/foods15152647)
Supplement: Supplementary file 1 [file foods-15-02647-s001.zip › foods-4426881-supplementary.pdf]

Dual-enzyme biocatalytic synthesis of novel rebaudioside M analogs with enhanced aqueous solubility, physicochemical stability, and sensory profiles

Hye-Jin Kang[a,#], Ok-Cheol Kim[b,#], Da-Eun Jang[b], Hee-Min Lee[c], Bu-Soo Park[d], Masayuki Okuyama[e],Jeong-Yong Cho[b], Seong-Jin Hong[a,*], Young-Min Kim[b,*]

[a] *Research Institute of Agricultural Science and Technology, Chonnam National University, Gwangju 61186, South Korea*

[b] *Department of Integrative Food, Bioscience and Biotechnology, Graduate School of Chonnam National University, Gwangju, 61186, South Korea*

[c] *Industrial Intelligence Research Division, World Institute of Kimchi, Gwangju 61755, Republic of Korea*

[d] *Food Biotech R&D Center, Samyang Corp., Seongnam-si, Republic of Korea*

[e] *Research Faculty of Agriculture, Hokkaido University, Sapporo 060-8589, Japan*

[#] These authors contributed equally to this work

**Corresponding author*

Seong-Jin Hong

E-mail address: hong0624243@naver.com (S.J. Hong), Tel.: +82-10-5755-4243

Young-Min Kim

E-mail address: u9897854@jnu.ac.kr (Y.M. Kim), Tel./Fax: +82-62-530-2142(2149)

Table S1. Comparison of representative enzymatic glucosylation strategies for steviol glycosides

**Glucosylation strategy**	**Enzyme source**	**Acceptor substrate**	**Major products**	**Key outcomes and limitations**	**Reference**
α-glucosylation	*Leuconostoc* species. Dextransucrase	Stevioside, Rebaudioside A	Glucosyl stevioside, Glucosyl rebaudioside A	Improved aqueous solubility and stability; did not generate RM-like glucosylated products	[15, 16, 17]
	Alkalihalobacillus oshimensis CGTase	Stevioside, Rebaudioside A	Glucosyl stevioside, Glucosyl rebaudioside A	Improved conversion efficiency; reduced sweetness intensity	[18]
β-glucosylation	*Stevia rebaudiana* UGTs (e.g. UGTSL2, UGT91D2, UGT76G1)	Stevioside, Rebaudioside A	Rebaudioside D, Rebaudioside M	Enabled efficient production of minor SGs; poor aqueous solubility	[8, 12]
Sequential α-/β-glucosylation	Leuconostoc kimchii Dextransucrase and S. rebaudiana UGT76G1	Stevioside	Rebaudioside M analog 1, Rebaudioside M analog 2	Higher aqueous solubility than RM, together with enhanced physicochemical stability	In this study

Table S2. ^{1}H- and ^{13}C-NMR data of RD2 in pyridine-d_5

Position	δ_H (*int.*, *mult.*, *J* in Hz)	δ_C	Position	δ_H (*int.*, *mult.*, *J* in Hz)	δ_C
1a<br>1b	1.76 (1H, d,12.0)<br>0.72 (1H, br. t, 12.0)	41.02	6′a<br>6′b	4.47 (1H, m)<br>4.19 (1H, m)	68.13
2a<br>2b	2.19 (1H, m)<br>1.39 (1H, d, 13.5)	19.79	1″	5.36 (1H, d, 3.5)	100.72
3a<br>3b	2.37 (1H, d, 13.5)<br>1.00 (1H, m)	38.73	2″	4.11	74.31
4	-	44.32	3″	4.57	75.74
5	1.01 (1H, br. d, 13.0)	57.70	4″	4.24	72.30
6a<br>6b	2.43 (1H, m)<br>1.93 (1H, m)	22.62	5″	4.49	74..48
7a<br>7b	1.46 (1H, m)<br>1.26 (1H, m)	41.97	6″a<br>6″b	4.47 (1H, m)<br>4.43 (1H, m)	63.03
8	-	42.58	1‴	5.05 (1H, d, 7.5)	97.80
9	0.87 (1H, d, 7.5)	54.70	2‴	4.06	84.52
10	-	40.16	3‴	4.46	78.50
11	1.65 (2H, m)	20.88	4‴	4.04	72.09
12a<br>12b	2.24 (1H, m)<br>1.94 (1H, m)	38.58	5‴	3.91	76.26
13	-	87.33	6‴a<br>6‴b	4.48 (1H, m)<br>4.33 (1H, m)	68.90
14a<br>14b	2.63 (1H, d, 11)<br>1.78 (1H, d, 11.5)	44.14	1⁗	5.24 (1H, d, 7.5)	106.91
15a<br>15b	2.08 (1H, d, 17)<br>1.97 (1H, d, 17.5)	48.45	2⁗	4.25	76.77
16	-	153.72	3⁗	4.21	78.70
17a<br>17b	5.77 (1H, s)<br>5.10 (1H, s)	105.84	4⁗	4.40	71.87
18	1.29(3H, s)	15.91	5⁗	3.96	78.79
19	-	177.37	6⁗a<br>6⁗b	4.54 (1H, m)<br>4.45 (1H, m)	63.30
20	1.27 (3H, s)	28.66	1″‴	5.51 (1H, d, 3.5)	100.76
1′	6.01 (1H, d, 7.5)	96.03	2″‴	4.19	74.26
2′	4.13	73.92	3″‴	4.64	75.61
3′	4.13	79.04	4″‴	4.22	72.52
4′	4.12	71.77	5″‴	4.47	74.37
5′	3.93	77.33	6″‴a<br>6″‴b	4.43 (1H, m)<br>4.36 (1H, m)	62.96

Table S3. ^{1}H- and ^{13}C- NMR data of RMLC1, *O*-β-D-glucosyl-(1″→3′)-*O*-α-D-glucosyl-(1‴→6′)-*O*-α-D-glucosyl-(1″″′→6″″)-stevioside

Position	δH (*int.*, *mult.*, *J* in Hz)	δC	Position	δH (*int.*, *mult.*, *J* in Hz)	δC
1a<br>1b	1.75 (1H, d, 12.0)<br>0.72 (1H, t, 12.0)	41.03	6′a<br>6′b	4.37 (1H, m)<br>4.12 (1H, m)	67.28
2a<br>2b	2.26 (1H, m)<br>1.40 (1H, m)	19.77	1″	5.34 (1H, d, 8.0)	105.27
3a<br>3b	2.26 (1H, d, 13)<br>0.99 (1H, m)	38.80	2″	4.03	74.20
4	-	44.32	3″	4.19	78.36
5	1.01 (1H, d, 11)	57.69	4″	4.09	71.97
6a<br>6b	2.33 (1H, m)<br>1.94 (1H, m)	22.42	5″	3.95	78.71
7a<br>7b	1.68 (1H, m)<br>1.28 (1H, m)	41.95	6‴a<br>6″b	4.50 (1H, m)<br>4.20 (1H, m)	62.77
8	-	42.61	1‴	5.32 (1H, d, 3.5)	100.48
9	0.87 (1H, m)	54.75	2‴	4.27	72.53
10	-	40.15	3‴	4.60	75.68
11	1.66 (2H, m)	20.85	4‴	4.04	74.10
12a<br>12b	2.36 (1H, m)<br>1.95 (1H, m)	38.70	5‴	4.51	74.44
13	-	87.30	6‴a<br>6‴b	4.48 (1H, m)<br>4.40 (1H, m)	63.00
14a<br>14b	2.57 (1H, d, 11)<br>1.89 (1H, d, 11)	43.80	1″″	5.04 (1H, d, 8.0)	97.95
15a<br>15b	2.20 (1H, d, 17.5)<br>2.00(1H, d, 17.5)	48.38	2″″	4.06	84.14
16	-	153.84	3″″	4.28	78.51
17a<br>17b	5.07 (1H, s)<br>5.72 (1H, s)	105.61	4″″	4.13	72.22
18	1.23 (3H, br. s)	15.85	5″″	4.01	76.15
19	-	177.3	6″″a<br>6″″b	4.47 (1H, m)<br>4.37 (1H, m)	69.06
20	1.24 (3H, br. s)	28.62	1″″′	5.25 (1H, d, 7.0)	106.79
1′	5.96 (1H, d, 8.0)	95.65	2″″′	4.20	76.72
2′	4.17	72.60	3″″′	4.20	78.60
3′	4.11	89.07	4″″′	4.54	72.47
4′	4.02	69.87	5″″′	4.02	78.80
5′	3.89	76.98	6″″′a<br>6″″′b	4.54 (1H, m)<br>4.43 (1H, m)	63.45

Position	δ_H (*int.*, *mult.*, *J* in Hz)	δ_C	Position	δ_H (*int.*, *mult.*, *J* in Hz)	δ_C
1′′′′′′	5.56 (1H, d, 3.5)	100.52			
2′′′′′′	4.16	74.22			
3′′′′′′	4.64	75.59			
4′′′′′′	4.12	74.48			
5′′′′′′	4.44	74.34			
6′′′′′′a<br>6′′′′′′b	4.43 (1H, m)<br>4.36 (1H, m)	63.00			

Table S4. ^{1}H- and ^{13}C- NMR data of RMLC2, *O*-β-D-glucosyl-(1″→3′)-*O*-α-D-glucosyl-(1‴→6′)-*O*-β-D-glucosyl-(1⁗′→3⁗)-*O*-α-D-glucosyl-(1⁗″→6⁗)-stevioside

**Position**	**δH (*int.*, *mult.*, *J* in Hz)**	**δC**	**Position**	**δH (*int.*, *mult.*, *J* in Hz)**	**δC**
1a<br>1b	1.77 (1H, d, 12.5)<br>0.72 (1H, t, 12.5)	41.01	6′a<br>6′b	4.26 (1H, m)	68.62
2a<br>2b	2.21 (1H, m)<br>1.41 (1H, m)	19.77	1″	5.26 (1H, d, 8.0)	104.93
3a<br>3b	2.36 (1H, d, 13)<br>0.97 (1H, m)	38.88	2″	3.97	75.78
4	-	44.34	3″	4.26	78.66
5	1.02 (1H, d, 12.5)	57.71	4″	4.08	71.98
6a<br>6b	2.56 (1H, m)<br>1.99 (1H, m)	22.43	5″	3.95	78.40
7a<br>7b	1.78 (1H, m)<br>1.30 (1H, m)	42.08	6‴a<br>6″b	4.50<br>4.39	62.97
8	-	42.43	1‴	5.48 (1H, d, 3.5)	100.37
9	0.88 (1H, m)	54.79	2‴	4.15	72.47
10	-	40.17	3‴	4.53	76.04
11	1.66 (2H, m)	20.78	4‴	4.05	74.04
12a<br>12b	2.30 (1H, m)<br>1.94 (1H, m)	38.82	5‴	4.51	74.22
13	-	87.72	6‴a<br>6‴b	4.48 (1H, m)<br>4.40 (1H, m)	63.06
14a<br>14b	2.56 (1H, d, 11)<br>1.94 (1H, d, 11)	43.72	1⁗	4.96 (1H, d, 8.0)	98.01
15a<br>15b	2.20 (1H, d, 17.5)<br>2.02 (1H, d, 17.5)	48.24	2⁗	4.20	80.52
16	-	153.39	3⁗	4.10	87.72
17a<br>17b	5.56 (1H, br. s)<br>5.34 (1H, br. s)	105.19	4⁗	3.77	70.74
18	1.27(3H, s)	15.94	5⁗	3.87	76.02
19	-	177.41	6⁗a<br>6⁗b	4.37 (1H, m)<br>4.11 (1H, m)	67.30
20	1.24 (3H, s)	28.60	1⁗′	5.51 (1H, d, 7.5)	104.62
1′	5.97 (1H, d, 8.0)	95.86	2⁗′	4.19	76.29
2′	4.22	72.57	3⁗′	4.20	79.00
3′	4.21	88.47	4⁗′	4.56	72.44
4′	4.01	69.85	5⁗′	3.86	78.40
5′	3.92	77.06	6⁗′a<br>6⁗′b	4.50 (1H, m)<br>4.34 (1H, m)	63.67

Position	δ_H (*int.*, *mult.*, *J* in Hz)	δ_C	Position	δ_H (*int.*, *mult.*, *J* in Hz)	δ_C
1′′′′′′	5.32 (1H, d, 8.0)	104.87			
2′′′′′′	4.12	74.36			
3′′′′′′	4.07	78.71			
4′′′′′′	4.27	72.23			
5′′′′′′	4.24	78.21			
6′′′′′′a<br>6′′′′′′b	4.54 (1H, m)<br>4.20 (1H, m)	62.71			
1′′′′′′′	5.31 (1H, d, 3.5)	100.37			
2′′′′′′′	4.02	74.07			
3′′′′′′′	4.60	75.83			
4′′′′′′′	4.12	74.43			
5′′′′′′′	4.46	74.07			
6′′′′′′′a<br>6′′′′′′′b	4.43 (1H, m)<br>4.36 (1H, m)	63.01			

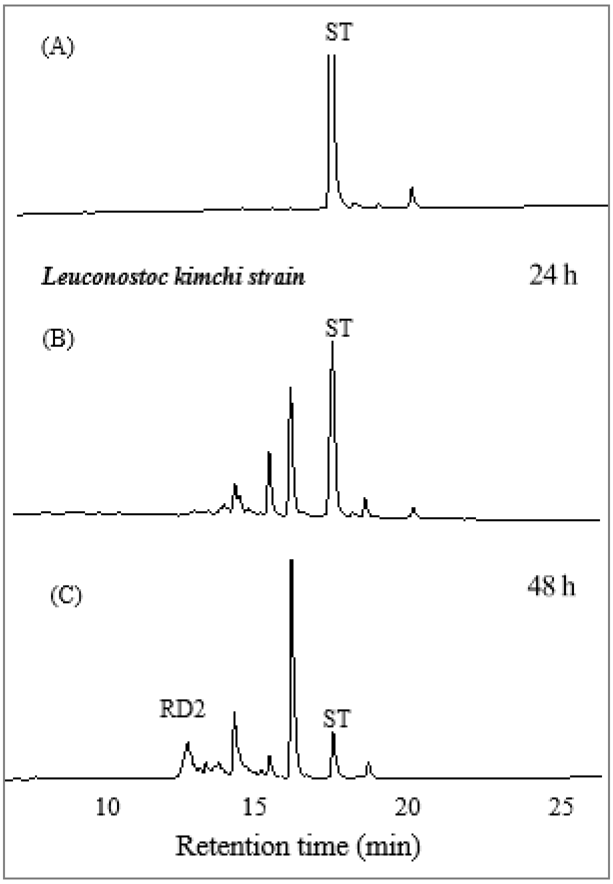

Figure S1. HPLC chromatograms of (A) ST standard, (B) the reaction mixture consisting of 40 mM sucrose, 20 mM ST, and 0.56 U/mL dextransucrase in 20 mM sodium acetate buffer (pH 5.5) was incubated at 30°C for 48 h, (C) the reaction mixture consisting of 40 mM sucrose, 20 mM ST, and 0.56 U/mL dextransucrase in 20 mM sodium acetate buffer (pH 5.5) was incubated at 30°C for 48 h. Abbreviaitons: ST: stevioside, RD2: Stev-G 3.1.

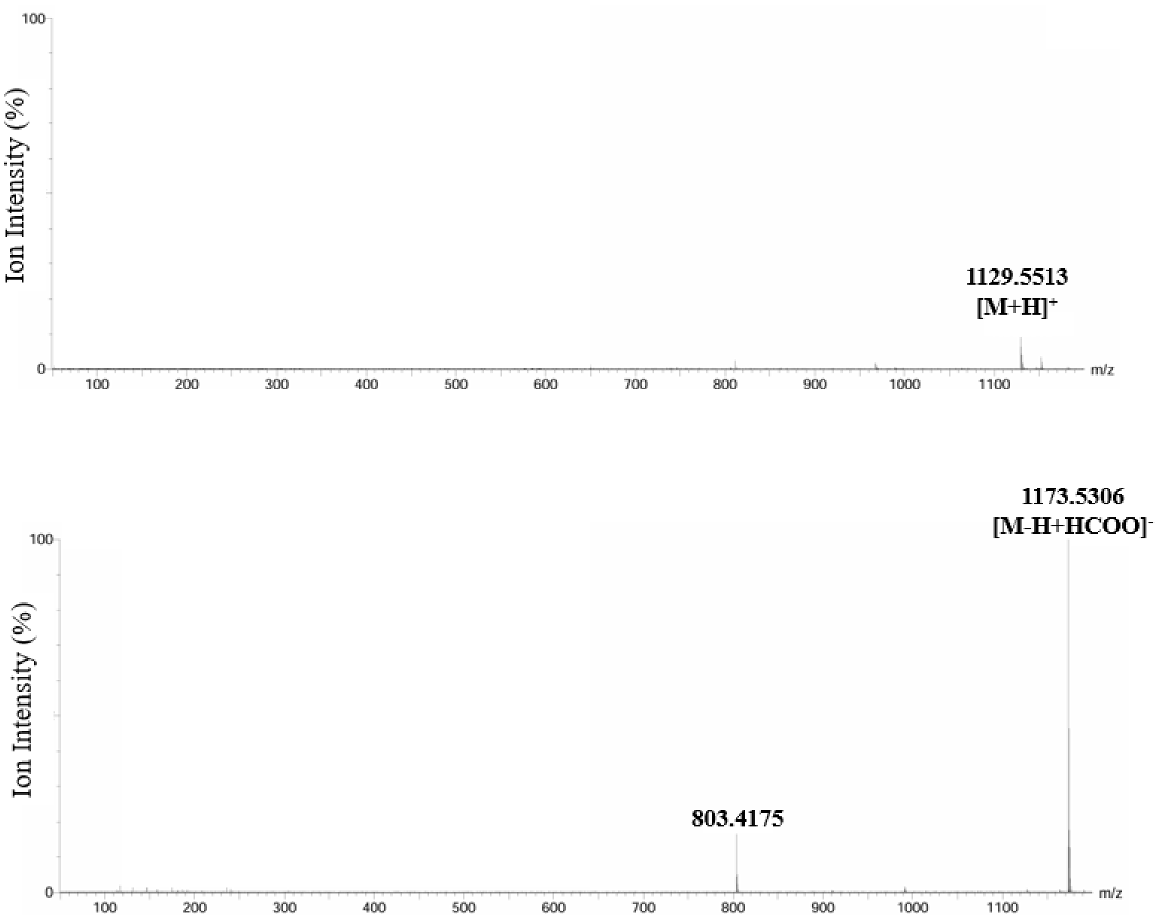

Figure S2. LC-ESI/MS analysis of the isolated RD2.

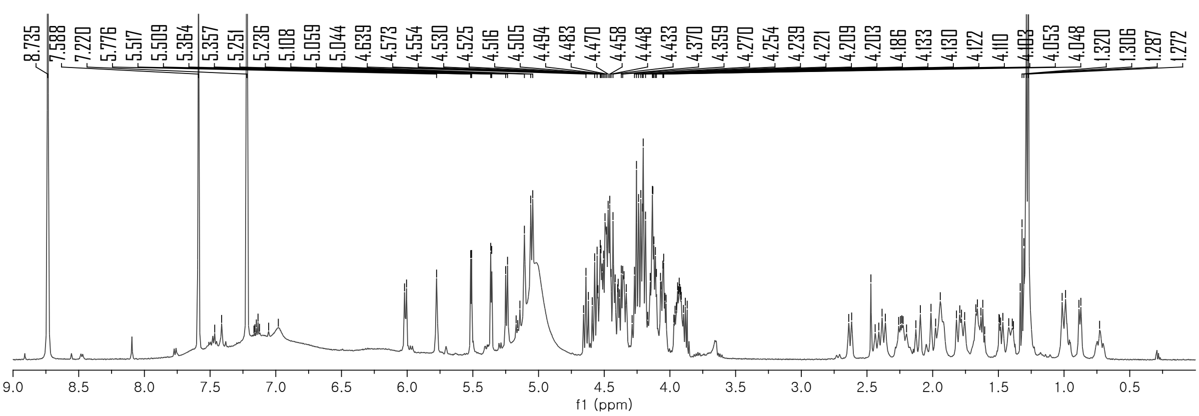

Figure S3. ^{1}H NMR (500 MHz) spectrum of RD2 in pyridin-d_5.

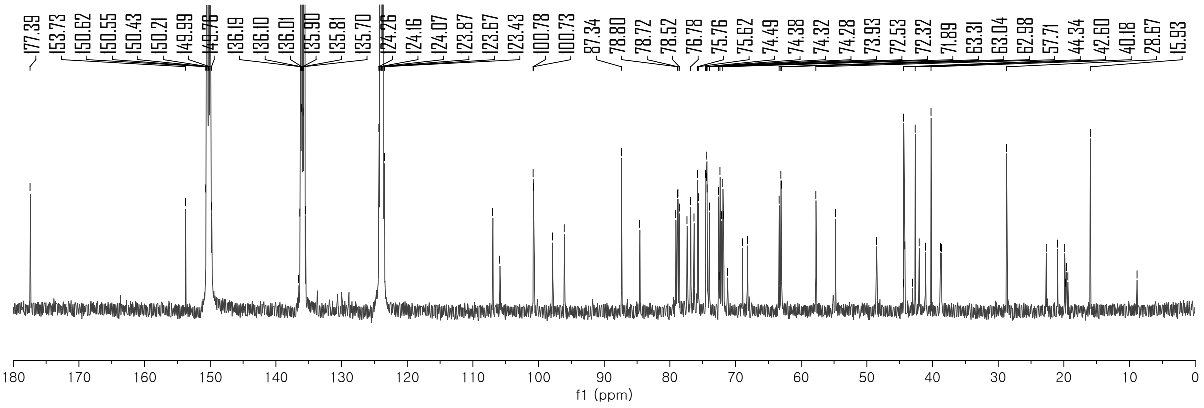

Figure S4. ^{13}C NMR (125 MHz) spectrum of RD2 in pyridin-*d*5.

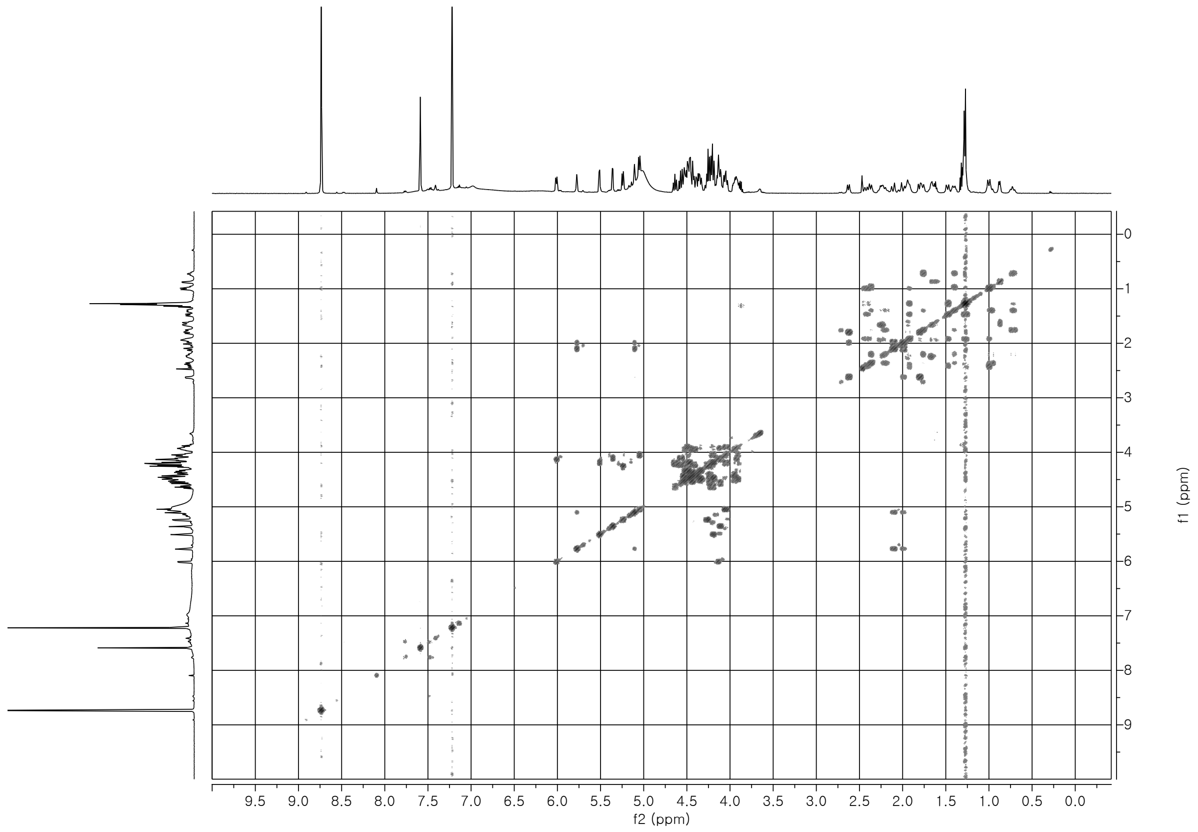

Figure S5. ^1H-^1H COSY spectrum of RD2.

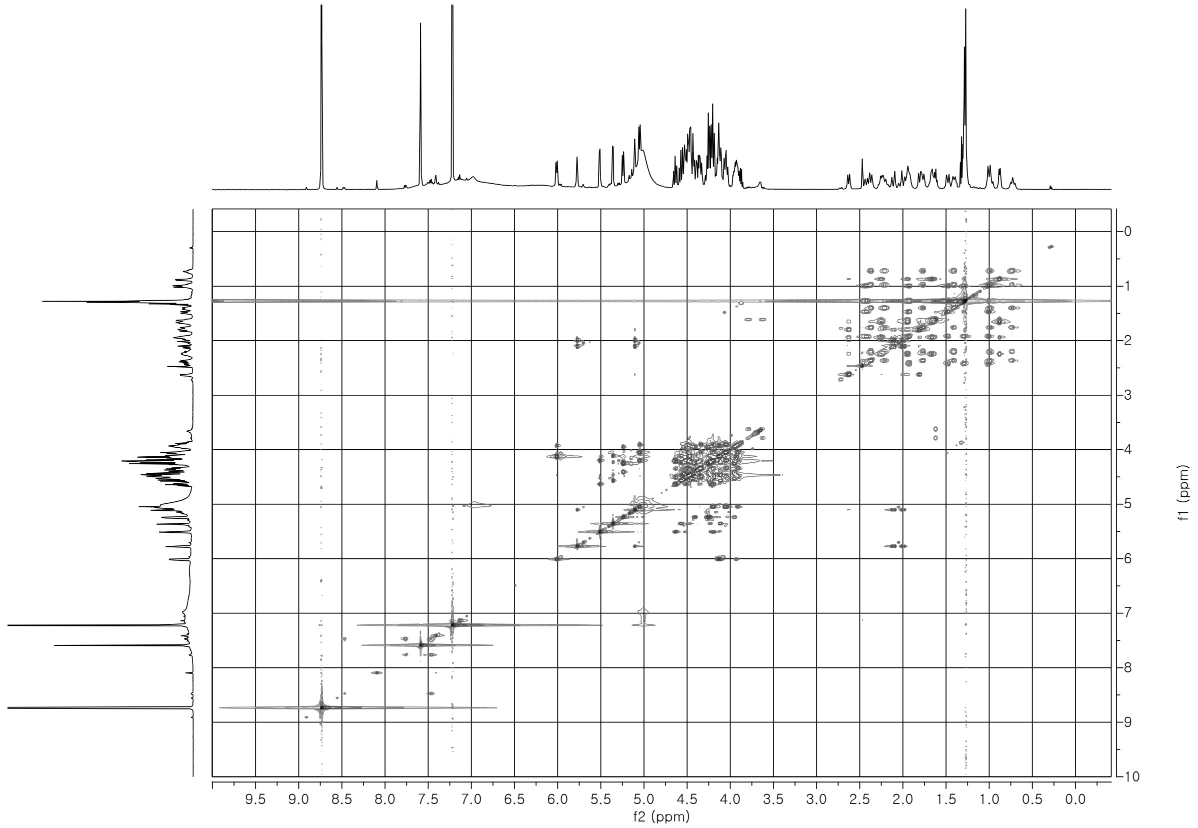

Figure S6. TOCSY spectrum of RD2.

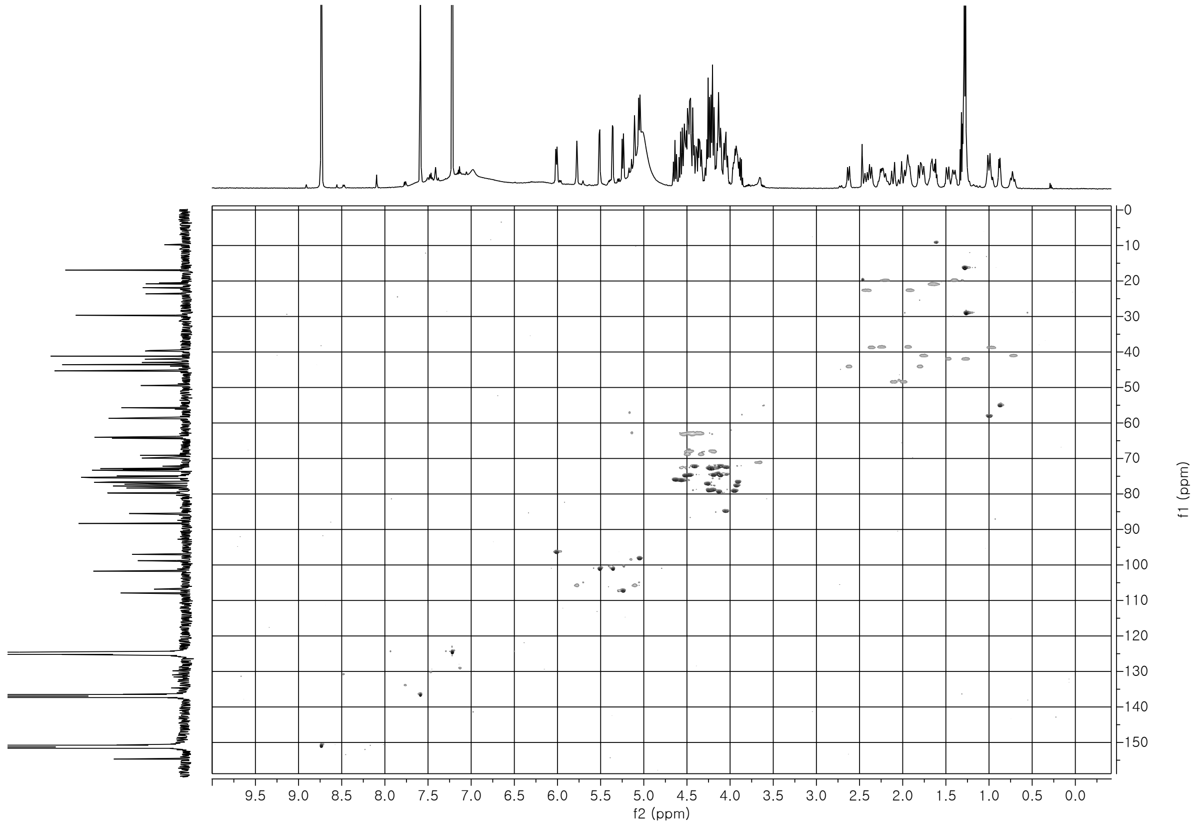

Figure S7. HSQC spectrum of RD2.

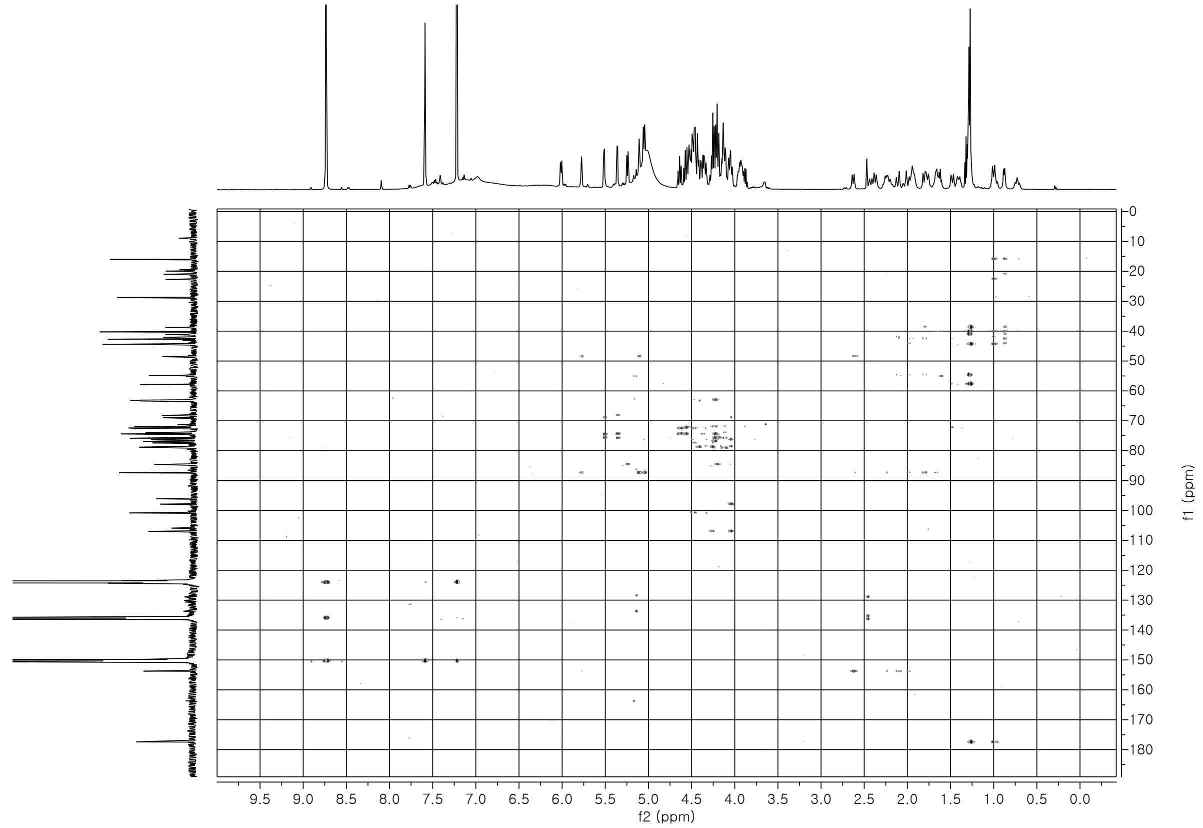

Figure S8. HMBC spectrum of RD2.

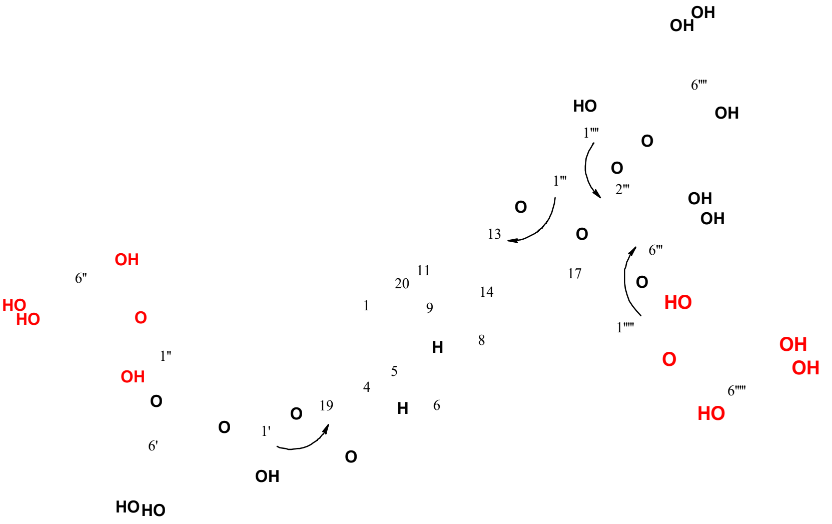

Figure S9. Structural analysis of RD2.

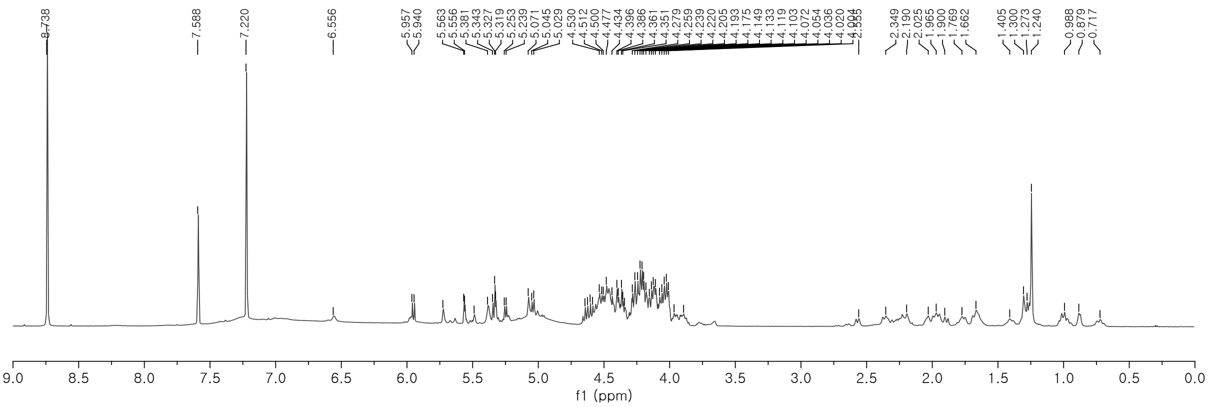

Figure S10. ^{1}H NMR (500 MHz) spectrum of rebaudioside M analog 1 (RMLC1) in pyridin-d_5.

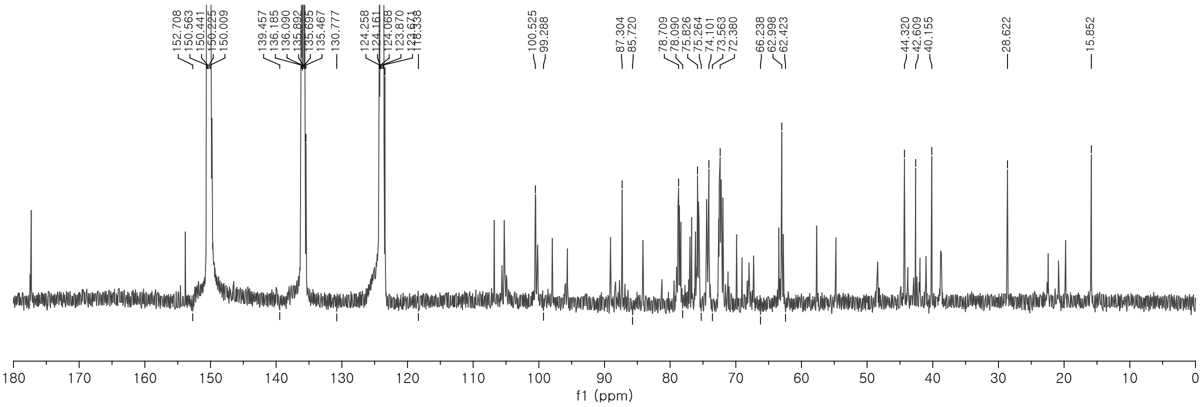

Figure S11. ^{13}C NMR (125 MHz) spectrum of rebaudioside M analog 1 (RMLC1) in pyridin-d_5.

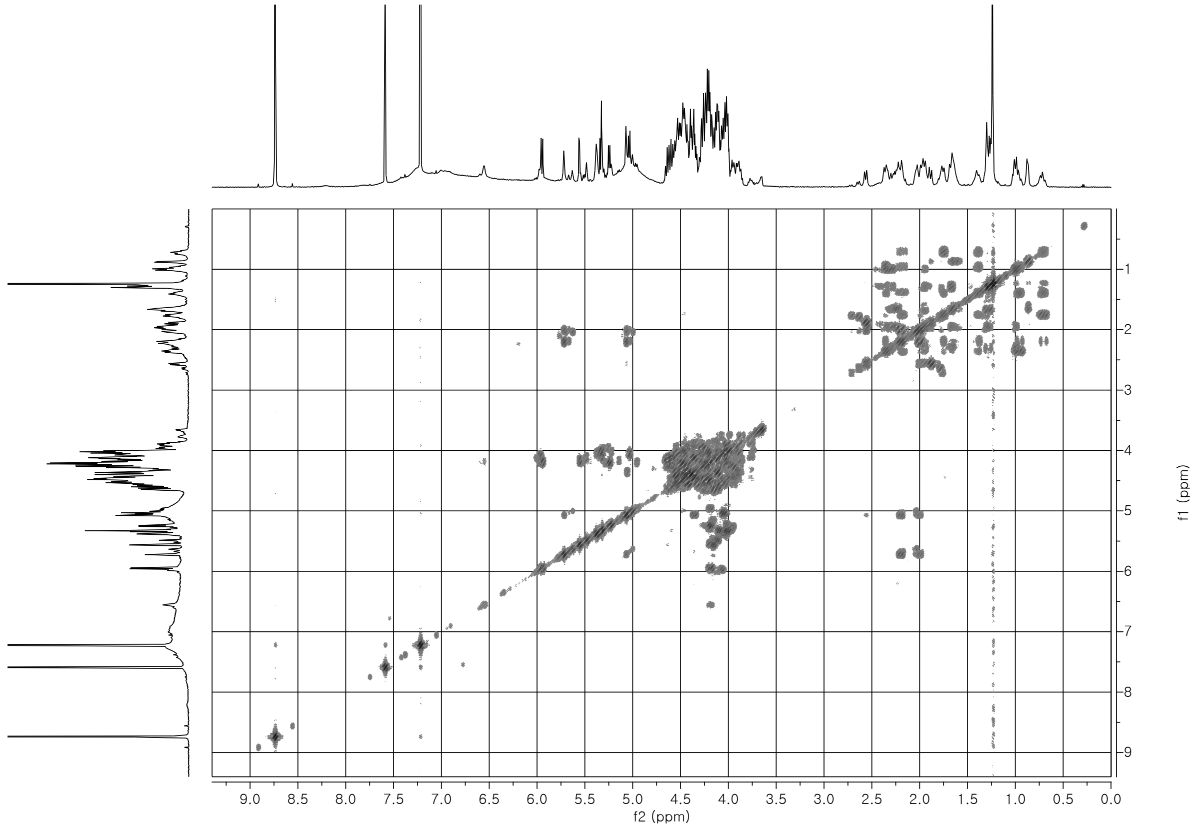

Figure S12. ^{1}H-^{1}H COSY spectrum of rebaudioside M analog 1 (RMLC1).

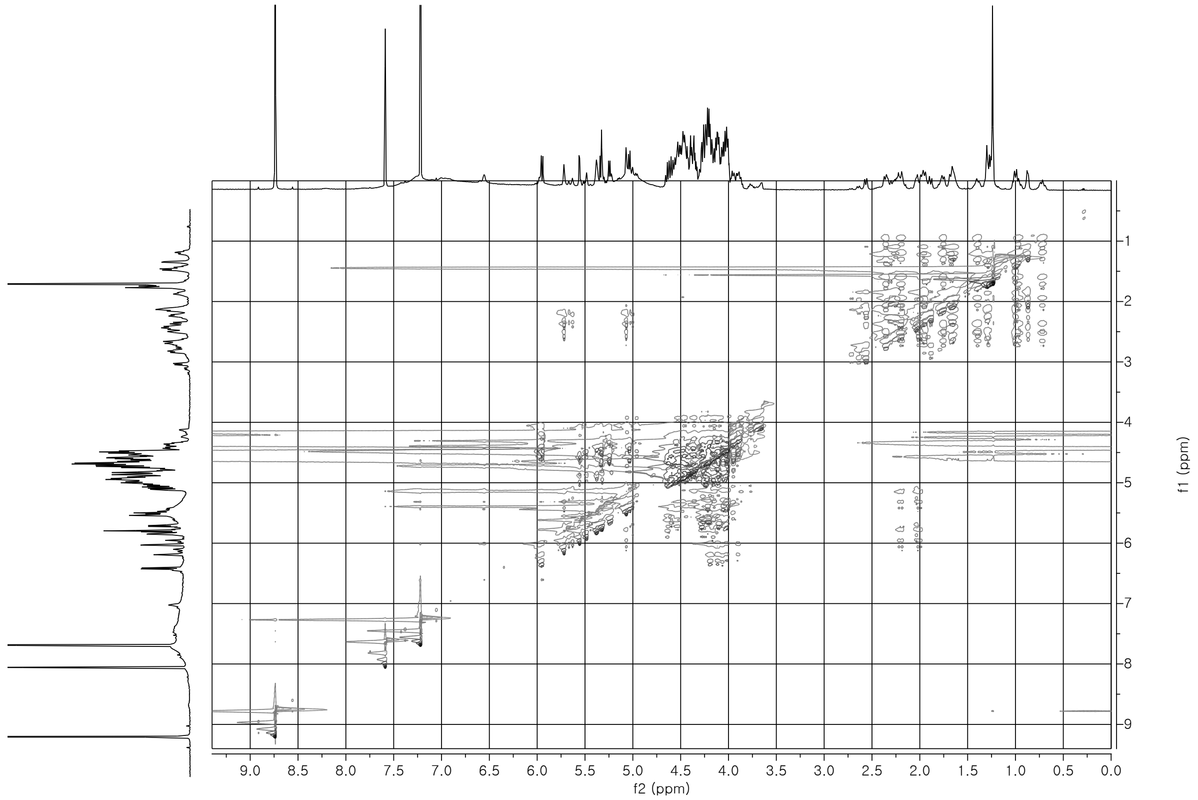

Figure S13. TOCSY spectrum of rebaudioside M analog 1 (RMLC1).

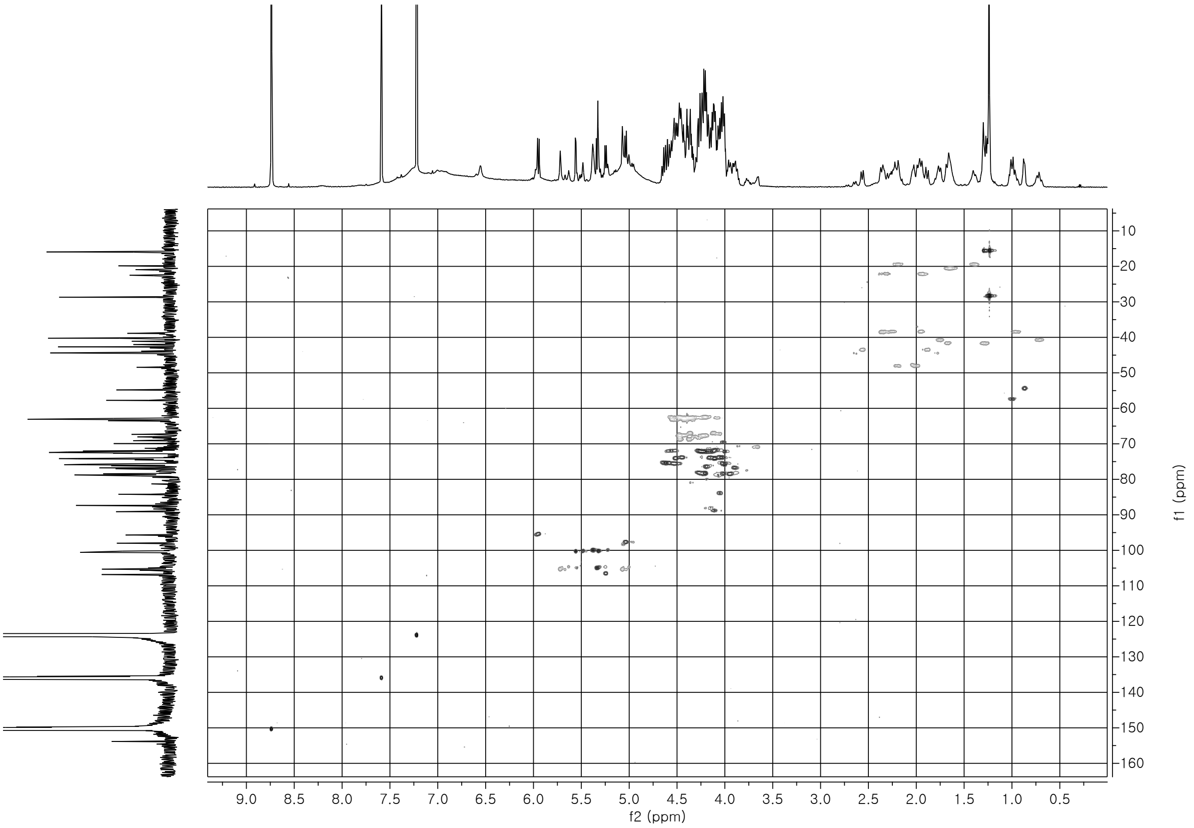

Figure S14. HSQC spectrum of rebaudioside M analog 1 (RMLC1).

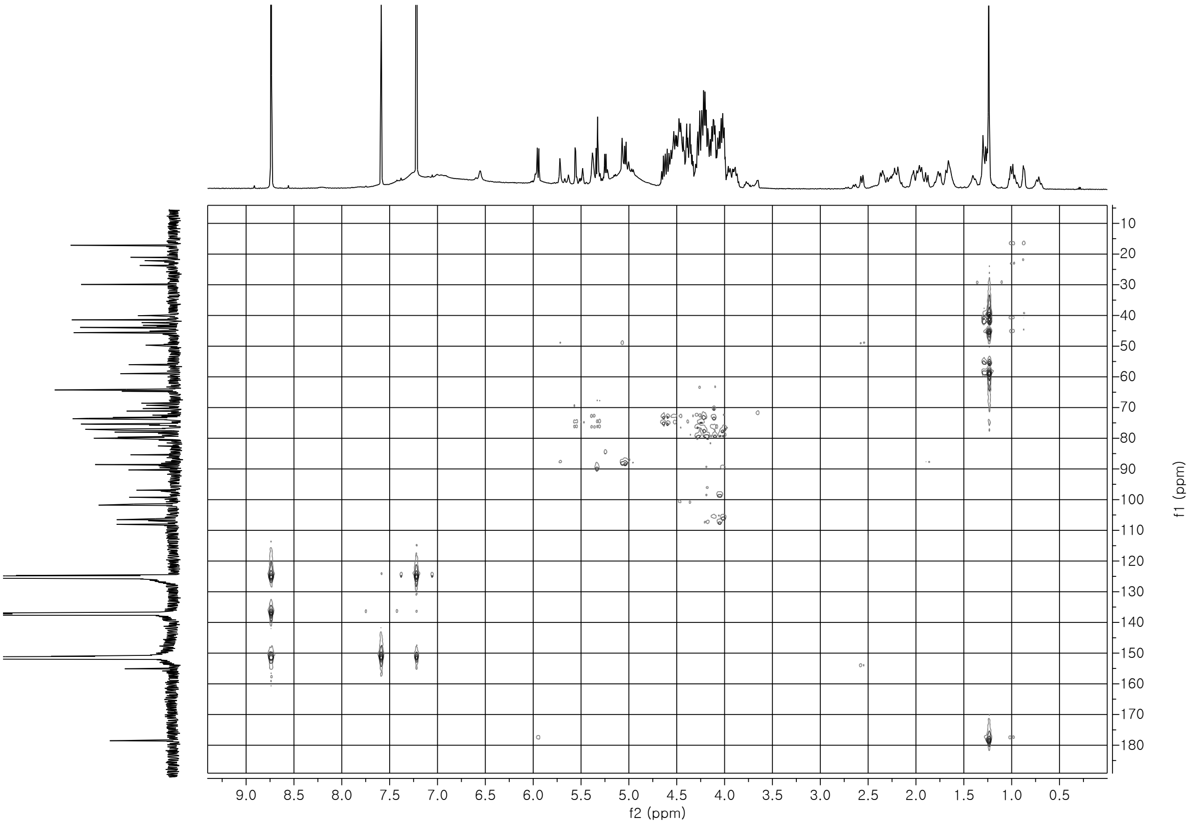

Figure S15. HMBC spectrum of rebaudioside M analog 1 (RMLC1).

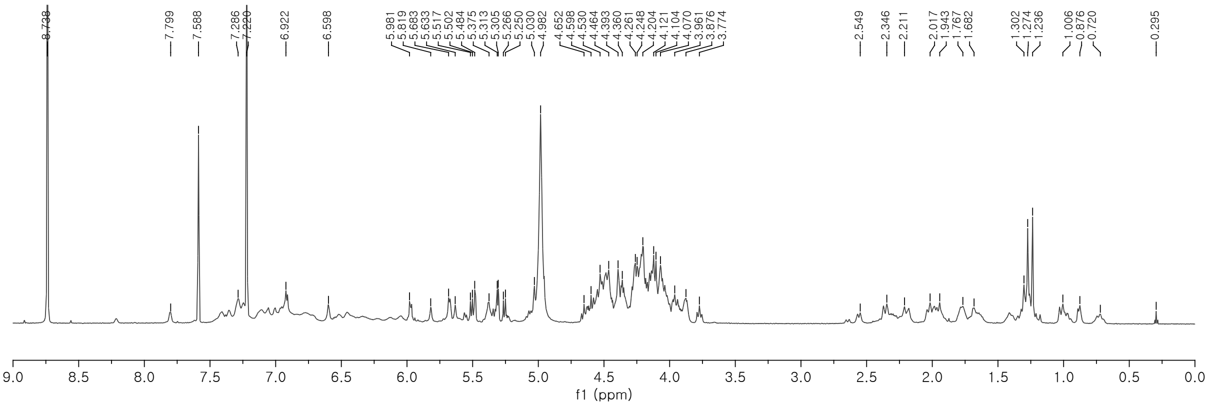

Figure S16. ^{1}H NMR (500 MHz) spectrum of rebaudioside M analog 2 (RMLC2) in pyridin-*d*5.

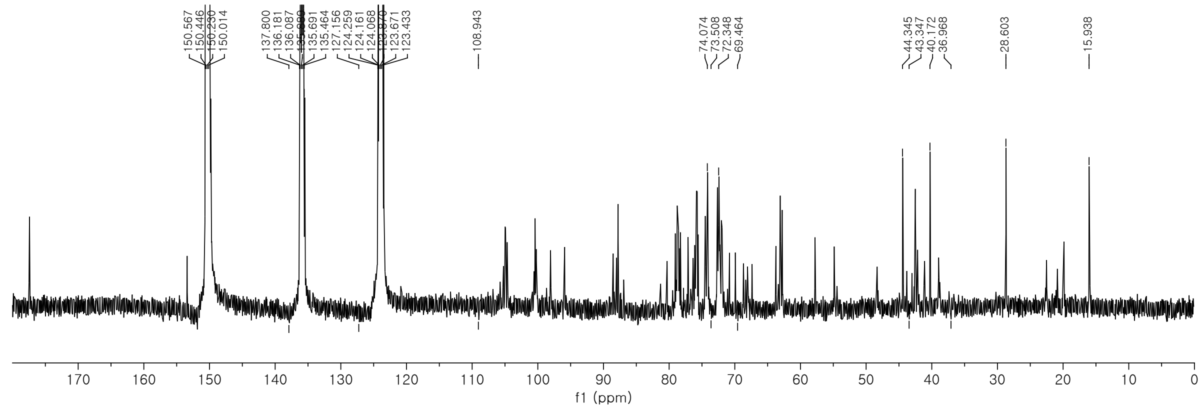

Figure S17. ^{13}C NMR (125 MHz) spectrum of rebaudioside M analog 2 (RMLC2) in pyridin-*d*5.

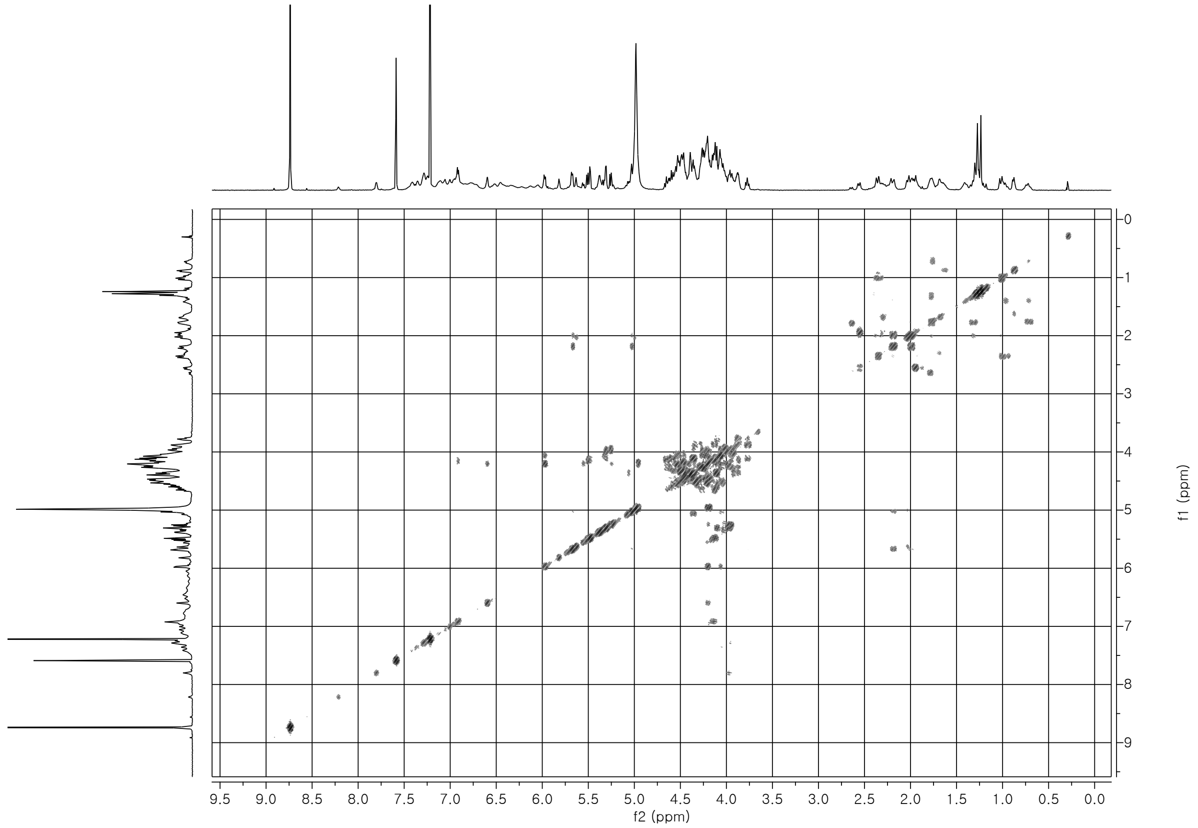

Figure S18. ^{1}H-^{1}H COSY spectrum of rebaudioside M analog 2 (RMLC2).

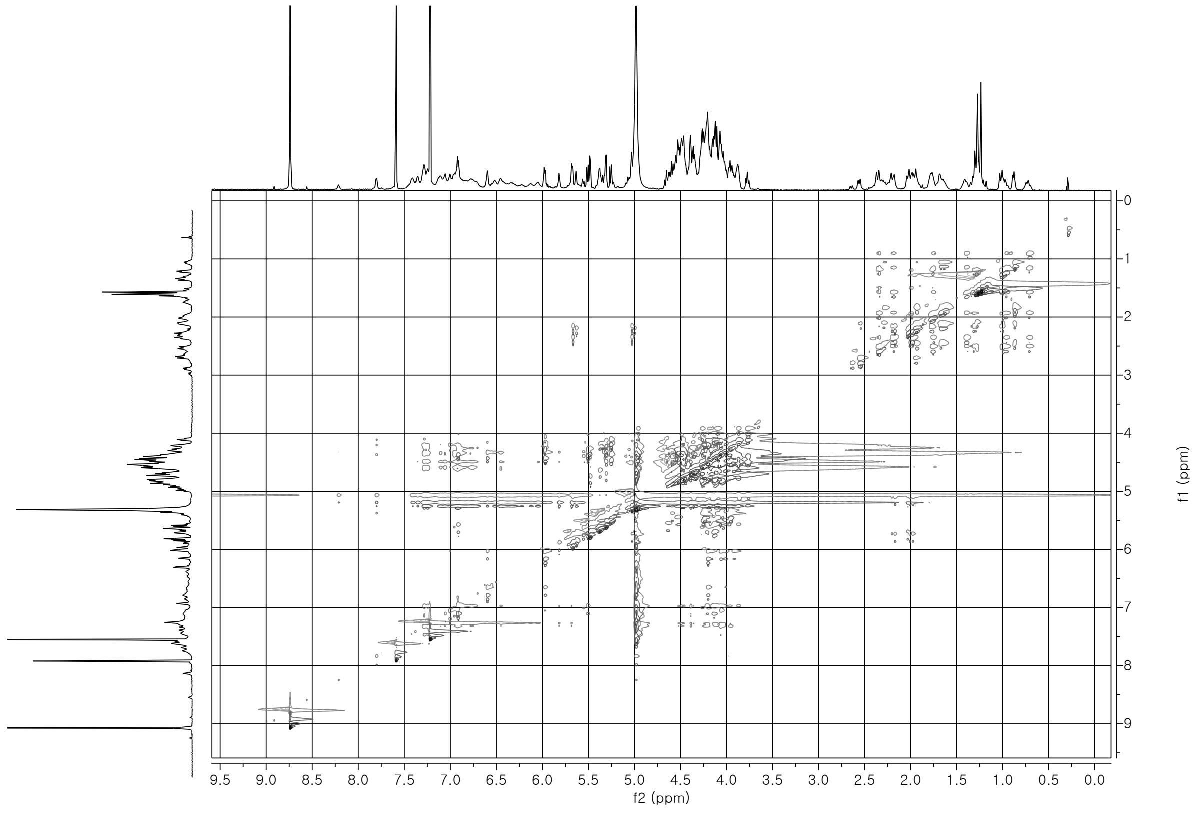

Figure S19. TOCSY spectrum of rebaudioside M analog 2 (RMLC2).

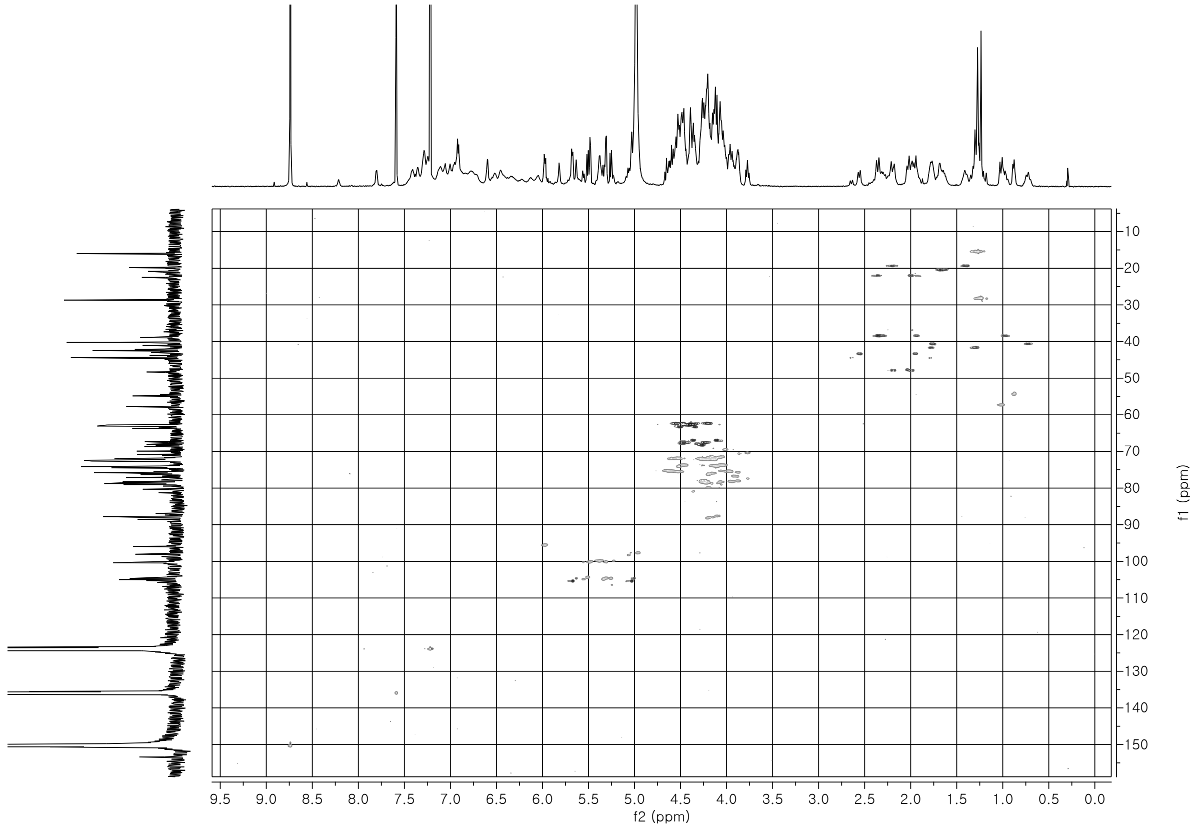

Figure S20. HSQC spectrum of rebaudioside M analog 2 (RMLC2).

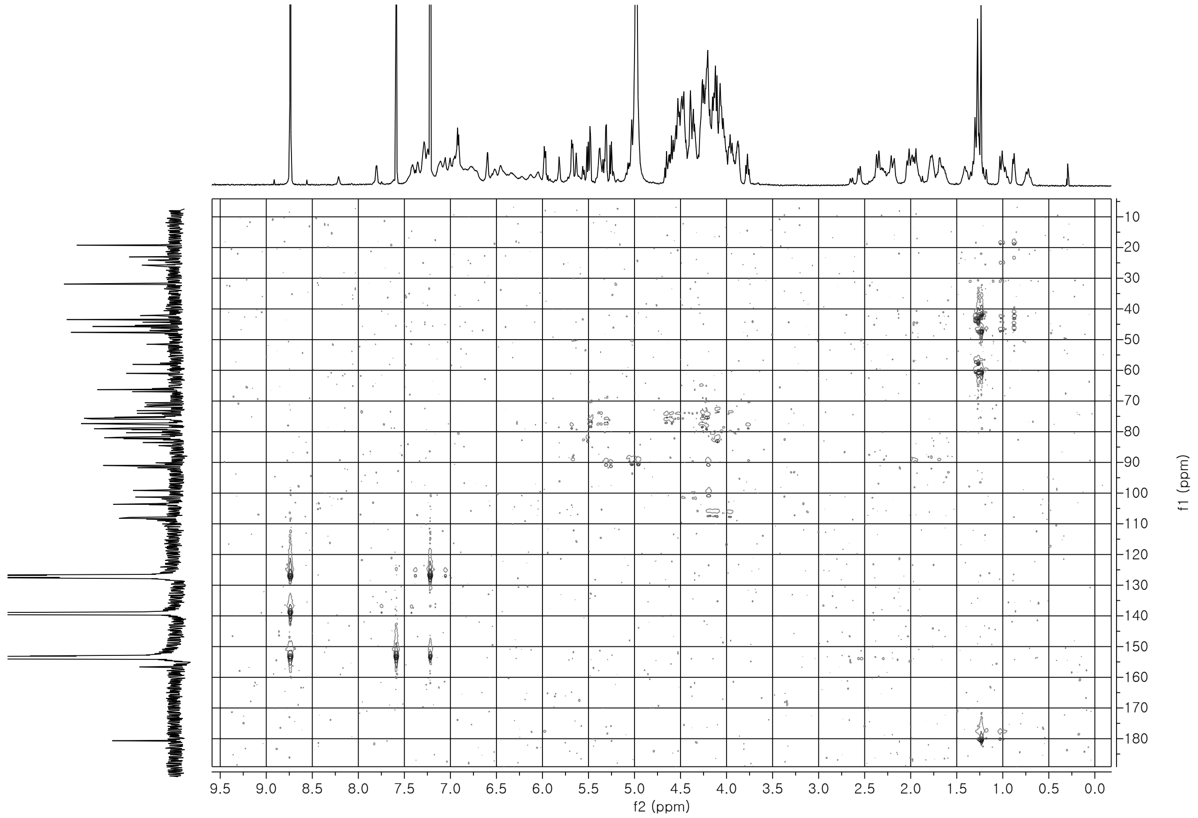

Figure S21. HMBC spectrum of rebaudioside M analog 2 (RMLC2).
